# Use of Clinical Isolates to Establish Criteria for a Mouse Model of Latent *Cryptococcus neoformans* Infection

**DOI:** 10.3389/fcimb.2021.804059

**Published:** 2022-02-02

**Authors:** Minna Ding, Kyle D. Smith, Darin L. Wiesner, Judith N. Nielsen, Katrina M. Jackson, Kirsten Nielsen

**Affiliations:** ^1^Department of Microbiology and Immunology, University of Minnesota Medical School, Minneapolis, MN, United States; ^2^Department of Pediatrics, University of Minnesota Medical School, Minneapolis, MN, United States; ^3^Department of Medicine, Center for Immunity and Inflammation, Rutgers New Jersey Medical School, Newark, NJ, United States; ^4^Department of Pathology and Laboratory Medicine, University of North Carolina, Chapel Hill, NC, United States

**Keywords:** *Cryptococcus neoformans*, cryptococcosis, cryptococcal meningitis, latent fungal infections, pulmonary granulomas, adaptive immunity, T-cells

## Abstract

The mechanisms of latency in the context of *C. neoformans* infection remain poorly understood. Two reasons for this gap in knowledge are: 1) the lack of standardized criteria for defining latent cryptococcosis in animal models and 2) limited genetic and immunological tools available for studying host parameters against *C. neoformans* in non-murine models of persistent infection. In this study, we defined criteria required for latency in *C. neoformans* infection models and used these criteria to develop a murine model of persistent *C. neoformans* infection using clinical isolates. We analyzed infections with two clinical *C. neoformans* strains, UgCl223 and UgCl552, isolated from advanced HIV patients with cryptococcal meningitis. Our data show that the majority of C57BL/6 mice infected with the clinical *C. neoformans* isolates had persistent, stable infections with low fungal burden, survived beyond 90 days-post infection, exhibited weight gain, had no clinical signs of disease, and had yeast cells contained within pulmonary granulomas with no generalized alveolar inflammation. Infected mice exhibited stable relative frequencies of pulmonary immune cells during the course of the infection. Upon CD4+ T-cell depletion, the CD4^DTR^ mice had significantly increased lung and brain fungal burden that resulted in lethal infection, indicating that CD4+ T-cells are important for control of the pulmonary infection and to prevent dissemination. Cells expressing the T_bet_ transcription factor were the predominant activated CD4 T-cell subset in the lungs during the latent infection. These T_bet_-expressing T-cells had decreased IFNγ production, which may have implications in the capacity of the cells to orchestrate the pulmonary immune response. Altogether, these results indicate that clinical *C. neoformans* isolates can establish a persistent controlled infection that meets most criteria for latency; highlighting the utility of this new mouse model system for studies of host immune responses that control *C. neoformans* infections.

## Introduction

*Cryptococcus neoformans* is an opportunistic fungal pathogen that causes life-threatening cryptococcal meningitis in immunocompromised individuals. It is the second most common HIV/AIDS-associated infection worldwide, leading to 15% of all AIDS-related mortality annually (Rajasingham et al., 2017). Following the inhalation of spores or desiccated yeast cells, *C. neoformans* establishes an initial pulmonary infection that is characterized by granuloma formation (Baker, 1976; Casadevall and Perfect, 1998; Giles et al., 2009). In healthy individuals, the initial *C. neoformans* pulmonary infection is thought to establish a latent infection within the lungs that does not manifest any clinical symptoms (Pirofski and Casadevall, 2020). However, in immunocompromised individuals, such as individuals with primary immunodeficiencies, HIV-induced CD4 T-cell loss, transplant-induced immune suppression, or immunodeficiency due to cancer chemotherapy, this latent infection is no longer able to be controlled and the pulmonary infection disseminates - ultimately progressing to fatal cryptococcal meningitis (Iseki et al., 1994; Winkelstein et al., 2003; Zonios et al., 2007; Scott-Algara et al., 2010; Vinh et al., 2010; Browne et al., 2012; Jarvis et al., 2013; Marchand et al., 2013; Rosen et al., 2013; Agrawal et al., 2017; Tenforde et al., 2017).

The concept of *C. neoformans* latent infections in healthy individuals is supported by epidemiological, genotyping, and serologic data. *C. neoformans* seropositivity is observed in healthy children as young as 2 years old, suggesting that initial *C. neoformans* infections likely occur during childhood (Abadi and Pirofski, 1999; Goldman et al., 2001). Evidence for dissemination of these latent *C. neoformans* infections comes from analysis of isolates present in immigrant populations. Immigrants presenting with HIV-associated cryptococcal meningitis that had lived in France for a median of 9 years were infected with *C. neoformans* isolates that are not commonly found in France, suggesting that these patients acquired their *C. neoformans* infection before immigrating to France (Garcia-Hermoso et al., 1999). Similarly, transplant patients with positive *C. neoformans* serology developed cryptococcosis earlier than patients with no serologic evidence of *C. neoformans* (Saha et al., 2007), again suggesting reactivation of latent *C. neoformans* infection in these seropositive transplant patients. Thus, there is sufficient evidence for latency in the context of *C. neoformans* infections.

The pathogenesis of latent *C. neoformans* infections is still not well understood; an intratracheal model of persistent cryptococcosis in healthy rats is currently the only model available for the study of latent infections. The rat infection model has not been extensively studied for a number of reasons (Goldman et al., 1994; Goldman et al., 2000; Dromer et al., 2010). First, the rat model has been only minimally used to study the host immune response to latent infection, primarily due to the lack of genetic and immunological tools when compared to those that are available in the more versatile mouse models. Second, the rat model uses the *C. deneoformans* ATCC 24067 (52D; serotype D) strain, a laboratory strain that undergoes genetic and phenotypic changes affecting virulence under standard laboratory conditions (Franzot et al., 1998). *C. deneoformans* only accounts for 4% of all infections caused by the pathogenic *Cryptococcus* species complex; whereas *C. neoformans* accounts for 95% of all infections (Maziarz and Perfect, 2016). Ideally, the development of a latent animal model of *C. neoformans* infection should use clinically relevant strains to recapitulate human disease (De Sousa et al., 2021).

Clear criteria for defining latent cryptococcosis in animal models is also needed. Seropositive individuals and healthy individuals with pulmonary cryptococcal granulomas typically show no clinical signs of disease. Yet, such a superficial classification may not be sufficient. For example, dogma established in other latent pulmonary infection models would require no extrapulmonary dissemination. However, cases of extrapulmonary dissemination to cervical lymph nodes are observed in immunocompetent individuals (Baker, 1976; Bao et al., 2013; Gurung et al., 2014) and the prostate is known to be a frequent extrapulmonary site for latent *C. neoformans* infection (Larsen, 1989). Even during latent *Mycobacterium tuberculosis* infections, which has some similarities to latent cryptococcosis (Pirofski and Casadevall, 2020), there are documented clinical cases of extrapulmonary dissemination (Barrios-Payán et al., 2012).

A mouse model of latent cryptococcosis would be an ideal model for studying the host control of pulmonary cryptococcosis due to the vast immunological and genetic resources available in this animal model (Bockamp et al., 2002; Van Der Weyden et al., 2002). However, establishing *C. neoformans* latency in healthy mice was historically challenging. Immunocompetent mice intranasally infected with the *C. neoformans* reference strain KN99α at the standardly used inoculum of 5x10^4^ colony forming units are unable to contain the pulmonary infection, leading to mortality by 19-25 days post-infection (Crabtree et al., 2012; Wiesner et al., 2015; Wiesner et al., 2016; Wiesner et al., 2017). This susceptibility to pulmonary infection in mice is also observed during high-dose intratracheal inoculation of *C. deneoformans* strain 52D (ATCC 24067), where less than 20% of mice survive to 100 days post-infection (Feldmesser and Casadevall, 1997). Nevertheless, we recently showed that the mouse inhalation model of *C. neoformans* infection can recapitulate strain-specific clinical outcomes in HIV patients with cryptococcal meningitis (Mukaremera et al., 2019). In particular, we identified clinical *C. neoformans* isolates that did not cause lethal infections in humans receiving standard antifungal drug regimens and also produced non-lethal infections in mice, suggesting that mice can establish persistent *C. neoformans* infections (Mukaremera et al., 2019).

Here, we present a low-dose inhalation model of *C. neoformans* infection in healthy mice using clinically relevant strains and show these persistent infections meet a set of proposed criteria for latency. Our data show that the infected mice had increased weight gain with no clinical signs of disease, maintained a stable pulmonary fungal burden, and contained the yeast cells within pulmonary granulomas with no generalized alveolar inflammation. Upon CD4 depletion, we observed a significant increase in lung and brain fungal burden – indicating that CD4 T-cells are necessary for controlling both the pulmonary infection and preventing dissemination. As expected based on previous studies (Wozniak and Levitz, 2010), T_bet_-expressing T_H_1 cells were the predominant effector CD4 T-cell subset generated during the latent infection. Surprisingly, these activated T-cells were deficient in their ability to produce IFNγ, perhaps providing a reason why the infection is not completely cleared.

## Materials and Methods

### Ethics Statement

All animal experiments were done in concordance with the Animal Welfare Act, U.S. federal law, and National Institutes of Health guidelines. Mice were handled in accordance with guidelines defined by the University of Minnesota Institutional Animal Care and Use Committee under protocol numbers 1507-32827A, 1806-36048A, and 2104-39016A.

### Mice

All mice used in this study were C57BL/6, or derived from a C57BL/6 background. Mice were housed in specific pathogen-free conditions. CD4-Cre (Lee et al., 2001) and homozygous iDTR (Buch et al., 2005) breeding pairs were purchased from Jackson Laboratory, and were crossed to generate CD4-Cre/iDTR, referred to as CD4^DTR^, mice (Buch et al., 2005). Systemic CD4 T-cell ablation in the CD4^DTR^ mice was achieved by an initial intraperitoneal injection of 1µg diphtheria toxin (DT; Sigma) and an additional 200ng DT every 4 days. Tbet-zsGreen mice (Zhu et al., 2012) were crossed with FoxP3-RFP mice (Wan and Flavell, 2005) to generate Tbet-zsGreen FoxP3-RFP mice and were kindly gifted by Marc Jenkins. Mice used for all experiments were 6-8 weeks of age; all controls were sex- and age-matched.

### Cryptococcus Strains

Cryptococcus strains KN99α (*C. neoformans*) (Nielsen et al., 2003), UgCl223 (*C. neoformans*) (Mukaremera et al., 2019), and UgCl552 (*C. neoformans*) (Mukaremera et al., 2019) were stored as -80°C glycerol stocks, streaked on yeast peptone dextrose (YPD) agar plates and incubated for 2 days at 30°C prior to use. YPD broth was inoculated with colonies from the aforementioned plate and incubated for 16 hours at 30°C and 225 RPM. The resulting inoculum was prepared by centrifuging the culture for 1 minute at 14,000 RPM (17,968xg) to pellet the cells, washing the cells 3 times with phosphate buffered saline (PBS), and resuspending the cells in PBS at a concentration of either 1x10^6^ cells/mL (KN99α) or 2x10^3^ cells/mL (UgCl223, UgCl552).

### Infection

A well-established intranasal pulmonary inhalation model of cryptococcosis was used for this study (Sabiiti et al., 2012). 6-8 week old, sex-matched mice were fully anesthetized with pentobarbital until mice did not respond to a toe pinch. 5x10^4^ (KN99α) or 1x10^2^ (UgCl223, UgCl552) *C. neoformans* cells in 50µL of PBS was placed on the nares of each mouse, and the mice inhaled the inoculum into the lower respiratory tract. The mice were suspended by their incisors for 5 minutes and subsequently paced upright on a paper towel in their cage until regaining consciousness. For survival studies, ten mice per group were infected as described above. Animals were monitored for morbidity and sacrificed when endpoint criteria were reached. Endpoint criteria were defined as 20% total body weight loss, loss of 2 grams of weight in 2 days, or symptoms of neurological disease. For organ fungal burden analysis, lungs, spleen, and brain were collected at the time of mouse sacrifice and placed into 2 mL sterile PBS. The collected tissues were homogenized, serial dilutions of tissue homogenates were plated on YPD agar plates supplemented with 0.04 mg/mL chloramphenicol and incubated at 30°C. *C. neoformans* colonies were counted after 48 hours of incubation.

### Antibody Depletion

For CD8 monoclonal antibody depletion studies, mice were intranasally infected with UgCl223 cells and treated with 15µg/g body weight of anti-CD8 monoclonal antibody (2.43; Bio X Cell) intraperitoneally at 28 days post infection for 3 consecutive days. A booster dose of 15µg/g anti-CD8 monoclonal antibody was also administered once every 7 days to ensure ≥98% depletion of CD8 T-cells in the lungs and spleen. The efficiency of CD8 T-cell depletion was assessed by flow cytometric analysis using an anti-CD8 antibody (53-6.7; Biolegend) that binds to a region of CD8 distinct from 2.43 (**Figure 7B**). Lung fungal burden was measured at 35- and 49-days post-infection.

For CD4 monoclonal antibody depletion studies, mice were intranasally infected with UgCl223 cells and treated with anti-CD4 monoclonal antibody (GK1.5; Bio X Cell) intraperitoneally at 28 days post infection for 3 consecutive days. A booster dose of anti-CD4 monoclonal antibody was also administered once every 3 days. We determined that a booster dose every 7 days was insufficient to maintain CD4 T-cell depletion (data not shown). Optimal CD4 T-cell depletion was determined by titrating CD4 monoclonal antibody concentration during infection with UgCl223, however only partial depletion (≤95%) was achieved for CD4 T-cells in the lungs (see **Supplementary Figure 2**). The efficiency of CD4 T-cell depletion was assessed by flow cytometric analysis using an anti-CD4 antibody (RM4-4; Biolegend) which binds to a region of CD4 distinct from GK1.5 (**Supplementary Figure 2**). An isotype antibody control (LTF-2; Bio X Cell) and PBS vehicle control was used in the CD8 and CD4 monoclonal antibody depletion experiments.

### Pulmonary Leukocyte Preparation

For analysis of the pulmonary immune response, we utilized intravascular staining to discriminate between vascular and tissue leukocytes as described previously (Anderson et al., 2014). Briefly, mice were intravenously injected with BUV395 (30-F11, BD Biosciences), APC-eFluor780 (30-F11, eBioscience), PerCp-Cy5.5 (30-F11, Biolegend), or APC (30-F11, Biolegend) labelled CD45 antibodies and were euthanized 3 minutes after injection with CO_2_. Pulmonary leukocytes were isolated as described previously (Yu et al., 2016). Briefly, the chest cavity was opened, and the trachea was exposed. Lungs were inflated with 2mL of digestion solution containing 1.5mg/mL Collagenase A (Roche), 5mM DNase I (Ambion), 5% fetal bovine serum (FBS), and 10mM HEPES in HBSS. Lungs were excised and placed in 5mL of digestion solution. Lung tissue plus digestion solution was incubated in a 37°C water bath for 30 minutes with gentle vortexing every 8-10 minutes. Upon completion of digestion, 25mL of PBS was added and the samples were vortexed at maximal speed for 30 seconds. The resulting cell suspensions were strained through a 70µm cell strainer. Cells were pelleted and resuspended in 40% Percol-RPMI medium (GE Life Sciences). A percol density gradient was created (40% top, 67% bottom) and the samples were centrifuged for 20 minutes at 650 x g. The leukocytes at the interface were removed, washed 2 times with 1mg/mL BSA and 0.002mM EDTA in PBS, and pelleted. Leukocytes were then treated with ACK RBS lysis solution, pelleted, and resuspended with PBS + FBS at a concentration of 10^7^ cells/mL. For analysis of T_H_ subsets and IFNγ expression studies, CD4 T-cells were isolated *via* negative selection using the EasySep™ Mouse CD4+ T Cell Isolation Kit (19852, StemCell) from single cell suspension of pulmonary leukocytes.

### IFNγ Restimulation Assay

Negatively selected CD4 T-cells isolated from Tbet-zsGreen FoxP3-RFP mice were stained with a Near-IR Live/Dead viability dye (Biolegend) according to manufacturer’s instructions. After staining, Tbet-zsGreen+ cells were captured using a BD FACSAria II cell sorter. The gating strategy started with doublet exclusion, then excluding dead cells, then excluding CD45+ IV-labelled cells, then excluding FoxP3-RFP+ cells, then gating on Tbet-zsGreen+ cells (**Supplementary Figure 5**).

Tbet-zsGreen+ cells or un-sorted negatively selected CD4 T-cells were re-suspended in RPMI-1540 (Life Technologies) supplemented with 10% fetal bovine serum (Gibco), 1% Penicillin/Streptomycin (Gibco), 100μM 2-mercaptoethanol (Sigma-Aldrich), and 1X L-glutamine (ThermoFisher Scientific) in 96-well round-bottom polypropylene tissue culture plates (Corning). A portion of cells were restimulated (*stimulated*) using a Cell Stimulation Cocktail (plus protein transport inhibitors, 00-4975-93, eBioscience) according to the manufacturer’s instructions. The remaining control (*unstimulated*) samples were treated with a Protein Transport Inhibitor Cocktail (00-4980-93, eBioscience). Samples were then incubated for 2 hours at 37°C in a CO_2_ incubator, washed, and stained for flow cytometric analysis.

### Flow Cytometry

All single-cell suspensions were stained with a Near-IR Live/Dead viability dye (Biolegend) according to manufacturer’s instructions and then incubated for 15 minutes with CD16/32 antibody (Biolegend) to prevent nonspecific antibody binding. For surface staining, samples were stained with fluorophore-labelled antibodies at 4°C for 30 minutes. If no additional processing was needed, then samples were washed, fixed with a 1:1 ratio of IC fixation buffer (eBioscience) to cell staining buffer (Biolegend), and stored at 4°C until ready for data acquisition by flow cytometry. For intracellular staining, cells were washed, fixed, and permeabilized using the Foxp3/Transcription Factor Staining Buffer Set (00-5523, eBioscience) according to the manufacturer’s instructions. Samples were stained intracellularly with fluorophore-labelled antibodies at 4°C overnight. After staining, cells were washed, placed in cell staining buffer, and stored at 4°C until ready for data acquisition by flow cytometry.

All data was acquired with a BD LSR Fortessa flow cytometer using BD FACSDiva software (BD Bioscience). Compensation was performed at the beginning of each experiment with UltraComp eBeads Plus Compensation Beads (Invitrogen) and an ArC Amine Reactive Compensation Bead Kit (Life Technologies). Data was analyzed using FlowJo v.10.6.1.

For pulmonary leukocyte response to *C. neoformans* infection, the following fluorophore-labelled antibodies were used: CD45 (30-F11, BV650, Biolegend), CD11b (M1/70, APC, BD Biosciences), CD11c (HL3, BV786, BD Biosciences), CD24 (M1/69, BV711, BD Biosciences), CD64 (X54-5/7.1, BV421, Biolegend), IA/IE (M5/114.15.2, BV605, BD Biosciences), Ly6C (HK1.4, PerCP-Cy5.5, eBioscience), Ly6G (1A8, APC-Cy7, BD Biosciences), and Siglec F (E50-2440, PE-CF594, BD Biosciences). The gating strategy for DCs, macrophages, monocytes, eosinophils, T-cells, B-cells, and NK cells is shown in **Supplementary Figure 1**.

For pulmonary CD4 and CD8 response to *C. neoformans* infection, the following fluorophore-labelled antibodies were used: B220 (RA3-6B2, APC-eFluor780, ThermoFisher Scientific), CD11c (N418, APC-eFluor780, ThermoFisher Scientific), CD11b (M1/70, APC-eFluor780, ThermoFisher Scientific), F4/80 (BM8, APC-eFluor780, ThermoFisher Scientific), NK1.1 (PK136, APC-eFluor780, eBioscience), CD3 (17A2, AF700, Biolegend), CD4 (GK1.5, AF488, ThermoFisher Scientific), CD8 (53-6.7, BV650, Biolegend), and CD44 (IM7, BV605, Biolegend). Cells were identified as the following: CD4 T-cells = B220-CD11c-CD11b-F4/80-, NK1.1-, CD3+, CD4+, CD8-, activated CD4+ T-cells = B220-CD11c-CD11b-F4/80-, NK1.1-, CD3+, CD4+, CD8-, CD44+, CD8 T-cells = B220-CD11c-CD11b-F4/80-, NK1.1-, CD3+, CD8+, CD4-, and activated CD8 T-cells = B220-CD11c-CD11b-F4/80-, NK1.1-, CD3+, CD8+, CD4-, CD44+.

To measure CD4 and CD8 depletion efficiency, the following fluorophore-labelled antibodies were used: CD3ϵ (145-2C11, eFluor450, ThermoFisher Scientific), TCRβ (H57-597, PE, ThermoFisher Scientific), CD4 (RM4-4, APC, Biolegend), and CD8 (53-6.7, AF700, ThermoFisher Scientific). Cells were identified as the following: CD4 T-cells = CD3ϵ+TCRβ+CD4+CD8- and CD8 T-cells = CD3ϵ+TCRβ+CD4-CD8+. Efficiency of CD4 or CD8 T-cell depletion was determined by calculating the percentage of CD4 or CD8 T-cells out of the grandparent gate (i.e., live singlet lymphocytes) using FlowJo.

To differentiate the T_H_ subsets during UgCl223 infection, the following fluorophore-labelled antibodies were used: B220 (RA3-6B2, APC-eFluor780, ThermoFisher Scientific), CD11c (N418, APC-eFluor780, ThermoFisher Scientific), CD11b (M1/70, APC-eFluor780, ThermoFisher Scientific), F4/80 (BM8, APC-eFluor780, ThermoFisher Scientific), NK1.1 (PK136, APC-eFluor780, eBioscience), CD3 (17A2, AF700, Biolegend), CD4 (GK1.5, AF488, ThermoFisher Scientific), CD8 (53-6.7, BV650, Biolegend), CD44 (IM7, BV605, Biolegend), T_bet_ (4B10, BV421, Biolegend), GATA3 (16E10A23, AF647, Biolegend), and Foxp3 (FJK-16S, PE, ThermoFisher Scientific). The gating strategy for T_H_1, T_H_2, T_REG_, and undefined cells is shown in **Supplementary Figure 4**. The CD4 T-cell subset proportions was calculated by dividing each CD4 subset by the total of T_H_1, T_H_2, T_REG_, and undefined cells.

To measure IFNγ production following PMA/ionomycin restimulation, the following fluorophore-labelled antibodies were used: B220 (RA3-6B2, APC-eFluor780, ThermoFisher Scientific), CD11c (N418, APC-eFluor780, ThermoFisher Scientific), CD11b (M1/70, APC-eFluor780, ThermoFisher Scientific), F4/80 (BM8, APC-eFluor780, ThermoFisher Scientific), NK1.1 (PK136, APC-eFluor780, eBioscience), CD3 (17A2, AF700, Biolegend), CD4 (GK1.5, AF488, ThermoFisher Scientific), CD8 (53-6.7, BV650, Biolegend), CD44 (IM7, BV605, Biolegend), and IFNγ antibody (XMG1.2, BV421, Biolegend). The gating strategy for IFNγ+ cells is shown in **Supplementary Figure 5**. The proportion of IFNγ cells were determined using FlowJo.

### Cryptococcal Antigen (CrAg) Lateral Flow Assay

Mouse blood was collected *via* the lateral saphenous vein into K2 EDTA blood collection tubes (BD Vacutainer). At least 100-250µL of blood was collected per mouse. All blood specimens were tested for CrAg positivity using either a non-quantitative CrAg lateral flow assay (IMMY) or a semi-quantitative CrAg lateral flow assay (IMMY). All CrAg LFAs were performed according to manufacturer’s instructions. To determine CrAg titers of blood specimens that were CrAg positive, serial dilutions were performed with a starting dilution of 1:5, followed by 1:2 serial dilutions. CrAg titers were recorded as the highest dilution that yielded a positive result.

### Lung Histology

Lungs from two mice at 14 days (KN99α) or two mice at each of 7, 35, 70, or 90 days (UgCl552, UgCl223) post-infection were inflated with 10% neutral buffered formalin (Thermo Fisher Scientific, Rockford, IL) and then the lung block was removed and fixed in additional 10% neutral buffered formalin. The entire lung block including the heart, trachea, and associated glands were then processed for paraffin embedding. The resulting paraffin embedded block containing the entire lung block was cut down until the majority of the lung was visible in a single section. Two 5µm sections were placed on a slide and stained with hematoxylin and eosin (H&E). Each section was examined in its entirety at multiple levels of magnification starting at 0.5x, with increased magnification up to 40x, to identify both individual *Cryptococcus* cells as well as various sizes of granulomas and the associated inflammatory cells.

### Statistics and Modeling

Statistical analysis was performed with GraphPad Prism 8 software (La Jolla, CA). Lung, spleen, and brain fungal burden was analyzed using a least squares regression model with Gaussian distribution. Kaplan-Meier survival curves were analyzed for statistical significance using low-rank testing. Power calculations were performed to assess appropriate sample size for all experiments. Data were analyzed using two-tailed t-test, one-way, or two-way ANOVA with Bonferroni adjustments for multiple comparisons. Mouse weights were analyzed using simple linear regression on mean weights per time point. *P-*values ≤ 0.05 were considered statistically significant. All data presented in this study are representative from a minimum of at least three independent experiments or biological replicates.

## Results

### Criteria for a Mouse Model of Latent *C. neoformans* Infection

Prior to developing a mouse model of *C. neoformans* latency, we formulated criteria for latency which recapitulate clinical findings by examining published literature on latent *C. neoformans* infections in humans. Individuals with latent *C. neoformans* infections do not have clinical symptoms, despite the continued presence of the fungus within the host (Dromer et al., 2010; Alanio, 2020; Pirofski and Casadevall, 2020) (**Table 1**). Autopsy studies suggest that *C. neoformans* persists within pulmonary granulomas during latent infection (Baker, 1976). From these same studies, it was inferred that the parenchyma surrounding the “small, circumscribed granulomatous lesions in the lung” have a healthy appearance (Baker, 1976) (**Table 1**). Beyond these few defining characteristics, there is a paucity of information concerning *C. neoformans* latency in humans. As such, we needed to rely on clinical manifestations and laboratory findings of cryptococcal meningitis to formulate additional criteria for latency. Diagnosis of cryptococcal meningitis is confirmed *via* detection of the capsular polysaccharide antigen (CrAg) or glucuronoxylomannan (GXM) in the blood, urine, or cerebrospinal fluid using a lateral flow assay (LFA) (Manabe et al., 2014; Vidal and Boulware, 2015; Drain et al., 2019). In contrast, individuals with latent *C. neoformans* infections are not expected to be CrAg positive (**Table 1**). Finally, the high morbidity and mortality rate of cryptococcal meningitis is not reflected in latent *C. neoformans* infections (Abassi et al., 2015; Rajasingham et al., 2017) (**Table 1**).

**Table 1:** Criteria for latent *C. neoformans* infection models.

Criteria for *C. neoformans* latency						
	*C. neoformans* latency in humans	Cryptococcal meningitis in humans	KN99α lethal infection in mice	52D infection in rats	52D infection in C57BL/6 mice	Clinical strain infection in C57BL/6 mice
**Stable fungal counts in lungs**	**ND**	**ND**	**✘**	✔	✔	✔
**Pulmonary granulomas**	✔	**✘**	**✘**	✔	✔	✔
**Predominantly normal lung parenchyma**	✔	**✘**	**✘**	✔	✔	✔
**Clinical manifestations of disease**	**✘**	✔	✔	**✘**	**✘**	**✘**
**Weight loss attributable to disease**	**✘**	✔	✔	**✘**	**✘**	**✘**
**CrAg positive in blood, CSF, or urine**	**✘**	✔	✔	**10 DPI**	✔	**70 DPI**
**Extrapulmonary dissemination**	✔	✔	✔	✔	✔	✔
**Mortality associated with disease**	**✘**	✔	✔	**✘**	✔	**✘**✔

ND, not determined; DPI, days post-infection.

Animal models of cryptococcosis were also used to help define the criteria for *C. neoformans* latency. In particular, the rat model of pulmonary cryptococcosis using *C. deneoformans* strain 52D is considered the best model to currently study latent *Cryptococcus* infection (Dromer et al., 2010). Persistent infections with *C. deneoformans* strain 52D can also occur in mice (Hoag et al., 1995; Huffnagle et al., 1998; Chen et al., 2008; Guillot et al., 2008). Both the mouse and rat persistent 52D infection models result in stable fungal burden over time, pulmonary granulomas with predominantly normal lung parenchyma, and no clinical manifestations or weight loss attributable to the infection (Curtis et al., 1994; Goldman et al., 1994; Chen and Casadevall, 1999; Goldman et al., 2000) (**Table 1**). Interestingly, serum GXM was detected as early as 10 days post-infection in the 52D rat infection model (Goldman et al., 1994) (**Table 1**). Serum GXM levels were detected in mice infected with 52D *via* intravenous injection (Mukherjee et al., 1994) (**Table 1**); to our knowledge these serum GXM studies have not been performed in mice infected with 52D *via* intranasal or intratracheal inhalation. Mice infected with 52D *via* intranasal inoculation typically succumb to the infection by 100 days post-infection (Feldmesser and Casadevall, 1997; Chen and Casadevall, 1999) (**Table 1**). The lethal KN99α infection model in C57BL/6 mice provides a useful contrast by which to evaluate persistent infection models for latency (Wiesner et al., 2015). The lethal infection model is analogous to cryptococcal meningitis in humans by all the following parameters: increasing lung fungal burden, high mortality, clinical symptoms of disease including meningitis and weight loss, positive serum CrAg LFA, and uncontrolled fungal growth in the lungs (i.e., lack of granulomas) (Baker and Haugen, 1955; Baker, 1976; Nielsen et al., 2003; Wiesner et al., 2015) (**Table 1**).

We also considered absence of extrapulmonary dissemination as a possible criterion for *C. neoformans* latency, but we ultimately did not include it for the following reasons: (1) Cases of extrapulmonary dissemination to cervical lymph nodes have been observed in immunocompetent patients (Baker, 1976; Bao et al., 2013; Gurung et al., 2014) and the prostate has also been identified as a potential extrapulmonary site for latent *C. neoformans* infection (Larsen, 1989) (**Table 1**). (2) Extrapulmonary dissemination was observed in the brain, spleen, and kidney as early as 7 days post-infection in the rat model (Goldman et al., 1994) (**Table 1**). (3) Finally, there are documented clinical cases of extrapulmonary dissemination in latent *Mycobacterium tuberculosis* infections, which have similarity to latent cryptococcosis (Barrios-Payán et al., 2012; Pirofski and Casadevall, 2020). Thus, there was no clinical or experimental basis to require the absence of systemic dissemination as a criterion for a latent *C. neoformans* infection animal model.

Based on these data from human *C. neoformans* infections and existing animal models, we developed the following set of criteria to define latency in persistent *C. neoformans* infection models: 1) stable fungal counts in the lungs throughout the entirety of the infection, 2) generation of pulmonary granulomas and no alveolar inflammation in the surrounding lung parenchyma, 3) no clinical signs of disease throughout the entirety of infection (i.e. animals behave and appear healthy), 4) no weight loss attributable to disease, 5) serum CrAg (GXM) LFA negative, and 6) no mortality associated with disease. These criteria formed the basis by which we determined how closely our mouse model of persistent *C. neoformans* infection using clinical isolates recapitulated latent infections in humans (**Table 1**).

### Intranasal Infection With *C. neoformans* Clinical Isolates Results in Stable Lung Fungal Burdens and No Clinical Manifestations of Disease

In a previous study, we found that *C. neoformans* clinical isolates UgCl223 and UgCl552 did not cause lethal infections in A/JCr mice, even at 150 days post-infection (Mukaremera et al., 2019). These mice maintained low fungal burdens, had limited systemic dissemination, and did not develop clinical symptoms of cryptococcal meningitis. This previous study suggested the mouse inhalation model could be used to investigate pulmonary *Cryptococcus* infection in the context of latent infections.

**Figure 1:**
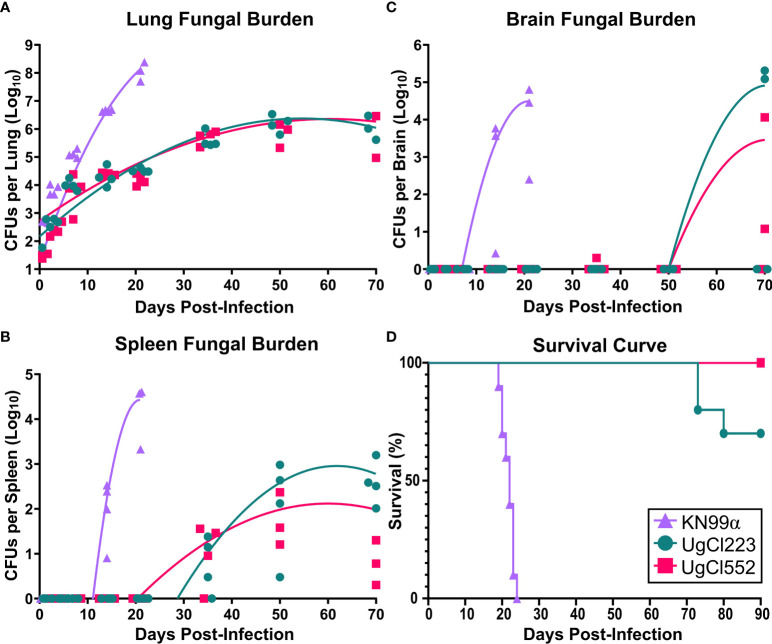
UgCl223 and UgCl552 infections had low fungal burdens and minimal mortality. C57BL/6 mice were intranasally infected with *C neoformans* strains KN99α (triangle), UgCl223 (circle), or UgCl552 (square). **(A–C)** UgCl223 and UgCl552 infections were characterized by low fungal burdens. **(A)** Lungs, **(B)** spleen, and **(C)** brain were harvested, homogenized, and plated for colony forming units (CFUs) at 0-, 3-, 7-, 14-, 21-, 35-, 50-, and 70-days post-infection. No animals selected for timepoints succumbed to the infection. No organs were collected for CFU analysis for KN99α infection at 35-, 50-, and 70-days post-infection because all mice succumbed to infection prior to these timepoints. Trendlines were obtained using least squares regression with Gaussian distribution. n = 3-4 mice per timepoint per strain. **(D)** The majority of mice infected with UgCl223 and UgCl552 remained healthy. Mice were monitored for signs of morbidity for 90 days and sacrificed at natural endpoint (20% total weight loss; 1 g/day weight loss for 2 consecutive days; or neurological symptoms including loss of sternal recumbency, partial paralysis, seizure, convulsion, or coma). n = 9-10 mice per strain.

**Figure 2:**
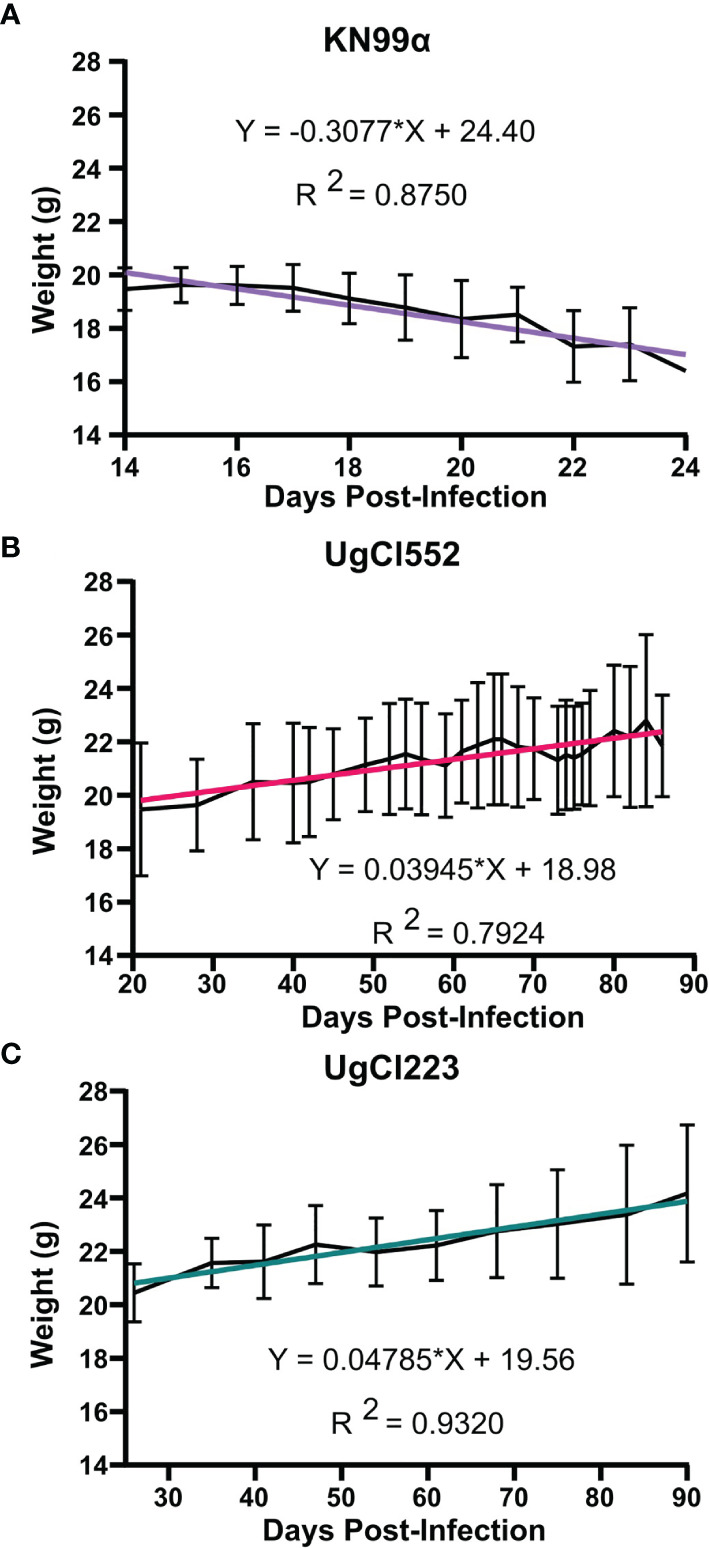
Mice infected with UgCl223 and UgCl552 maintained steady weight gain throughout the course of the infection. C57BL/6 mice were intranasally infected with **(A)** KN99α, **(B)** UgCl552, **(C)** UgCl223. Mouse weights in grams (g) were tracked until 90 days post-infection for UgCl552 and UgCl223, and 24 days post-infection for KN99α. Simple linear regression analysis was performed between mean weight of mice (n = 10) and days post-infection.

To further develop a possible mouse model of latent infection, we analyzed infections with these clinical isolates in the commonly utilized C57BL/6 inbred mouse background. To mimic a biologically relevant route of infection, we used a low inoculum dose of 1 x 10^2^ yeast cells provided *via* inhalation. Infection with either UgCl552 or UgCl223 established a controlled pulmonary infection in C57BL/6 mice characterized by persistent and stable low colony forming units (CFUs) in the lungs (**Figure 1A**). Low levels of dissemination to the spleen were also observed by 35 days post-infection, and all mice had detectable CFUs in the spleen by 70 days post-infection (**Figure 1B**). We observed low levels of dissemination to the brain with both strains; each strain had detectable CFUs in the brain at 70 days post-infection in only half of the mice (**Figure 1C**). Importantly, all the mice infected with UgCl552, and the majority of mice infected with UgCl223, exhibited no signs of disease, and remained healthy for at least 90 days post-infection (**Figure 1D**). Currently, we have UgCl223-infected mice that have survived up to 270 days post-infection. In addition, infected mice maintained steady weight gain throughout the entirety of the infection (**Figure 2A, B**). These findings are in contrast to mice infected with KN99α that developed an acute lethal infection, with increasing pulmonary CFUs (**Figure 1A**), robust systemic dissemination observed by 14 days post-infection (**Figure 1B, C**), steady weight loss (**Figure 2C**), and complete mortality of all mice within 25 days post-infection (**Figure 1D**). Unlike the persistent rat infection model which detected serum GXM by 10 days post-infection (Goldman et al., 1994), mice infected with UgCl223 had negative CrAg titers *via* LFA up to 35 days post-infection and low CrAg titers were only observed at 70 days post-infection (**Table 2**). Thus, while some systemic dissemination to the spleen and brain was observed, infected mice did not exhibit any clinical symptoms, had low serum CrAg titers only at late time-points, and the majority of mice did not succumb to the infection.

**Table 2:** UgCl223-infected mice were CrAg negative until 70 days post-infection.

*C. neoformans* Strain	Days Post-Infection	Total # of Mice	% CrAg Negative	% CrAg Positive	CrAg Titers
**Uninfected**	N/A	5	100%	0%	N/A, N/A, N/A, N/A, N/A
**KN99α**	14	2	0%	100%	1:5, 1:10
**KN99α**	19	1	0%	100%	1:2560*
**UgCl223**	7	3	100%	0%	N/A, N/A, N/A
**UgCl223**	14	8	100%	0%	N/A, N/A, N/A, N/A, N/A, N/A, N/A, N/A
**UgCl223**	24	3	100%	0%	N/A, N/A, N/A
**UgCl223**	35	3	100%	0%	N/A, N/A, N/A
**UgCl223**	70	3	67%	33%	N/A, N/A, 1:2
**UgCl223**	111	3	0%	100%	1:5, 1:5, 1:20^†^
**UgCl223**	128	1	0%	100%	1:2
**UgCl223**	149	2	0%	100%	1:2, 1:5
**UgCl223**	200	5	20%	80%	N/A, 1:2, 1:2, 1:2, 1:20^‡^

*Mouse was sacrificed due to clinical meningitis symptoms (endpoint criteria).

†Mouse showed no clinical meningitis symptoms and was healthy at time of blood draw.

‡Mouse survived to 207 days post-infection and was euthanized due to a non-C. neoformans lesion on the hind leg. N/A not applicable.

### Infection With *C. neoformans* Clinical Isolates UgCl223 and UgCl552 Results in Granulomas

**Figure 3:**
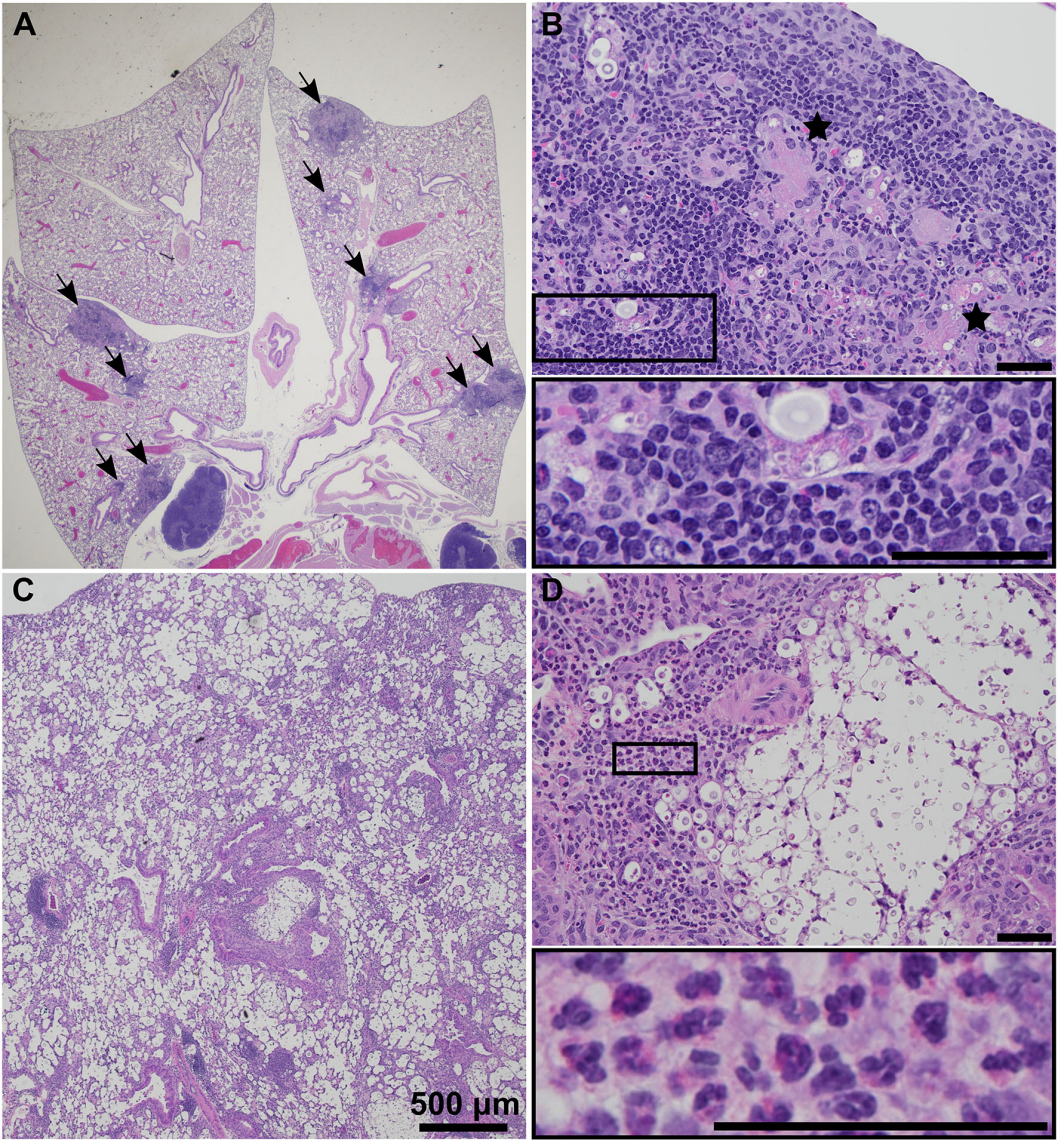
UgCl552 infection was contained within pulmonary granulomas. Representative histopathology images of granuloma formation at **(A)** 0.5x and **(B)** 20x magnification in lungs of a C57BL/6 mouse at 35 days post-infection with UgCl552. Black arrows point to granulomas. Black stars mark multinucleate giant cells. **(C)** 2x and **(D)** 20x magnification of lungs of a C57BL/6 mouse at 14 days post-infection with KN99α illustrating *C. neoformans* cells and inflammatory infiltrates with PMN’s visible in the insert. Scale bars = 50 μm, unless otherwise noted.

The pathology of both UgCl223 and UgCl552 infections in C57BL/6 mice was characterized by discrete granulomas multifocally within the pulmonary parenchyma (**Figure 3A**). The vast majority of the lung parenchyma was normal, with the exception of parenchyma immediately surrounding the granulomas that had variable low numbers of inflammatory infiltrates. Granulomas were visible by 14 days post-infection and changes in granuloma morphology were observed up to 70 days post-infection. *C. neoformans* cells were contained within the granulomas and localized intracellularly within macrophages or extracellularly within the larger granulomatous structure (**Figure 3B**). The inflammatory cells associated with the granulomas consisted of lymphocytes, epithelioid cells, multinucleate giant cells, and macrophages (**Figure 3B**). Of note, fewer polymorphonuclear cells (PMNs) were associated with cryptococci during UgCl552 infection, compared to lethal KN99α infection (**Figures 3C, D**). In the lethal KN99α infection, the cryptococci, with or without associated inflammatory cells, were disseminated throughout the pulmonary parenchyma (**Figure 3C**). Significant numbers of polymorphonuclear cells (PMNs), as well as some lymphocytes, were associated with large *C. neoformans* cell aggregations (**Figure 3D**). Overall, the lung histopathology findings from infection with the clinical isolates closely resembles lung autopsy findings from immunocompetent individuals (Baker, 1976), whereas the KN99α infection resembles lung autopsy findings from immunocompromised individuals with HIV that have acute cryptococcosis (Shibuya et al., 2005).

### Lung Immune Response to Persistent *C. neoformans* Infection Remains Stable Over Time

**Figure 4:**
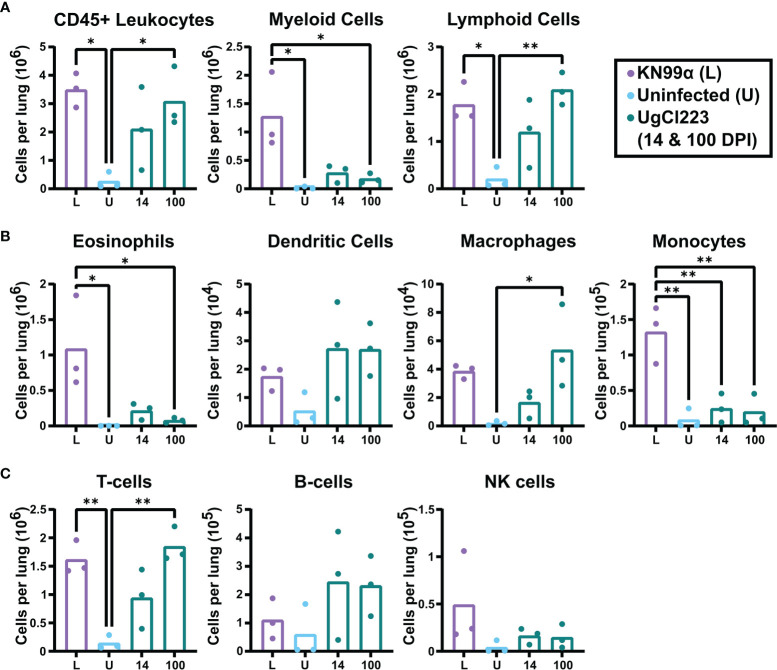
Lymphoid cells were the predominant immune cell in the lungs of UgCl223-infected mice. C57BL/6 mice were intranasally infected with UgCl223 or KN99α (lethal infection). For UgCl223, single cell lung suspensions were generated at 14- and 100-days post-infection. For KN99α, single cell lung suspensions were generated at 14-days post-infection. **(A)** Leukocyte, **(B)** lymphocyte, and **(C)** myeloid cell populations were quantified using flow cytometry. L = KN99α lethal infection at 14-days post-infection; U = uninfected control; 14 = UgCl223 infection at 14-days post-infection; 100 = UgCl223 infection at 100-days post-infection. The gating strategy is shown in **Supplementary Figure 1**. In brief, cells were identified as the following: Eosinophils = CD11b+CD24+SiglecF+, Dendritic Cells = CD11c+CD11b+/-CD24+MHCII+CD24-, Macrophages = CD64+CD24-, Monocytes = CD11b+Ly6C+/-MHCII-, T-cells = CD11b-CD11c-CD24-MHCII-, B-cells = CD11b-CD11c-MHCII+CD24^int^, NK cells = CD11b+CD64-MHCII-Ly6C-. n = 3 mice per group. *p < 0.05, **p < 0.001 by one-way ANOVA with Bonferroni correction.

The histopathology findings indicated that the host immune response in the lungs is different in UgCl223 and UgCl552 infections compared to the lethal KN99α infection. To further quantify the pulmonary immune response, we performed flow cytometry on single cell suspensions of infected mouse lungs (see **Supplemental Figure 1** for gating strategy). At 14 days post-infection, the number of total lung leukocytes in mice infected with UgCl223 was not significantly higher than the uninfected control mice, and lower than in mice infected with KN99α (**Figure 4A**). However, a significant increase in lung leukocytes was observed by 100 days post-infection in mice infected with UgCl223 (**Figure 4A**). This is most likely attributed to a significant increase in lymphoid cell numbers, as the number of myeloid cells did not increase between 14- and 100-days post-infection (**Figure 4A**).

Further analysis of the myeloid cell population revealed similar recruitment of eosinophils, dendritic cells, and monocytes in the UgCl223 infection at 14- and 100-days post-infection, and these were not significantly different from uninfected controls (**Figure 4B**). Although, there was a significant increase in macrophages compared to uninfected controls at 100-days post-UgCl223 infection (**Figure 4B**). Most notably, the UgCl223 infection did not result in the pulmonary eosinophil recruitment that is a hallmark of the lethal KN99α infection (Wiesner et al., 2015) (**Figure 4B**).

Analysis of the lymphoid population showed recruitment of T-cells to the lungs in the UgCl223 infection was significantly higher at 100 days post-infection compared to uninfected controls, but B-cell and NK cell numbers did not increase (**Figure 4C**). A significant increase in T-cells was also observed in the lethal KN99α at 14 days post-infection compared to uninfected controls (**Figure 4C**). Overall, these findings suggest that the increase in pulmonary CD45+ leukocytes during UgCl223 infection is attributed to increased T-cell recruitment to the site of infection.

### CD4 T-Cell Depletion During Persistent UgCl223 Infection Contribute to Mortality

**Figure 5:**
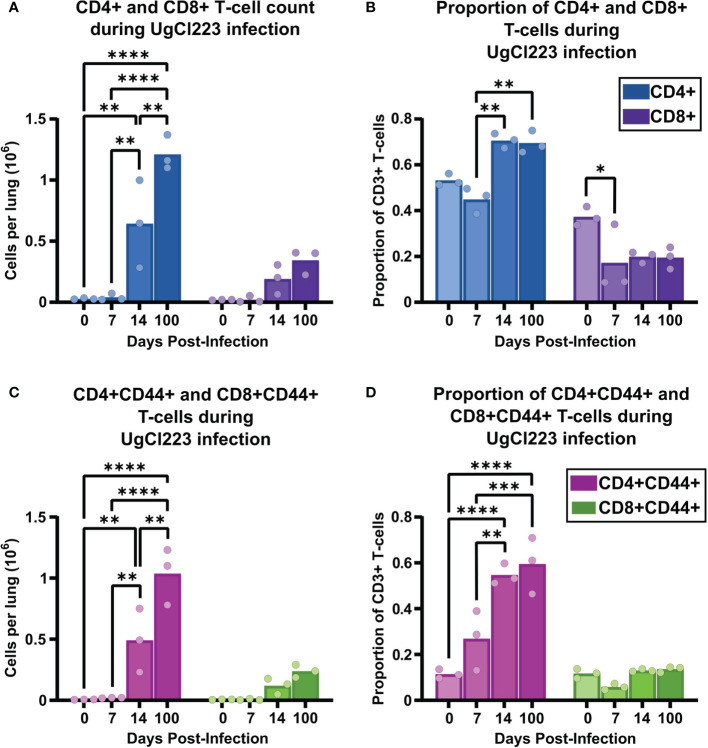
The adaptive immune response to UgCl223 infection within the lungs was dominated by CD4 T-cells. C57BL/6 mice were intranasally infected with UgCl223. Single cell lung suspensions were generated at 0, 7-, 14-, and 100-days post-infection. **(A)** Total CD4+ and CD8+ T-cell counts, **(B)** Total CD4+ and CD8+ T-cell proportion out of total CD3+ T-cells, **(C)** CD4+CD44+ and CD8+CD44+ activated T-cell counts, and **(D)** CD4+CD44+ and CD8+CD44+ activated T-cell proportion out of total CD3+ T-cells were quantified using flow cytometry. The gating strategy of the flow cytometric plots was doublet exclusion, exclusion of B220+CD11c+CD11b+F4/80+NK1.1+/dead cells/CD45 IV-labelled cells, gating on CD3+ T-cells, gating on CD4+ and CD8+ T-cells, and then gating on CD4+CD44+ and CD8+CD44+ T-cells. n = 3 mice per group. *p < 0.05, **p < 0.001, ***p < 0.0001, ****p < 0.00001 by two-way ANOVA. with Bonferroni correction. Statistical significance was noted between CD4+ vs. CD8+ groups, and CD4+CD44+ vs. CD8+CD44+ groups, but is not indicated on the graph.

The increased recruitment of T-cells to the lungs from 14- to 100-days post-infection during UgCl223 infection led us to hypothesize that the adaptive immune response, not the innate immune response, was critical for controlling lung fungal burden in the UgCl223 infections. This hypothesis was supported not only by clinical data regarding populations of patients acutely susceptible to cryptococcosis (Iseki et al., 1994; Winkelstein et al., 2003; Zonios et al., 2007; Scott-Algara et al., 2010; Vinh et al., 2010; Browne et al., 2012; Jarvis et al., 2013; Marchand et al., 2013; Rosen et al., 2013; Agrawal et al., 2017; Tenforde et al., 2017), but also earlier studies in mice using clearance models (Huffnagle et al., 1991; Huffnagle et al., 1994). In particular, we wanted to determine whether the increase in T-cells during UgCl223 infection was attributable to either CD4 or CD8 T-cells. We first analyzed recruitment of CD4 and CD8 T-cells to the lungs of mice infected with UgCl223. Both the number and proportion of CD4 T-cells were significantly higher compared to CD8 T-cells throughout the course of the infection (**Figure 5**). The total CD4 T-cell count increased significantly from 14- to 100-days post-infection (**Figure 5A**). We also observed a significant increase in the number of CD4+CD44+ activated T-cells (**Figure 5C**). However, the proportion of total CD4 T-cells and CD4+CD44+ activated T-cells out of total CD3+ T-cells did not change from 14- to 100- days post-infection (**Figures 5B, D****)**.

Interestingly, while we observed a trend towards increasing numbers of CD8 T-cells and CD8+ CD44+ activated T-cells from 14- to 100-days post infection in the lungs, the increase did not reach significance (**Figures 5A, C****)**. In addition, there was no change, nor trend towards increased CD8 T-cell and activated CD8+ CD44+ T-cell proportions compared to the uninfected control (**Figures 5B, D****)**. Consistent with the previous studies and anecdotal observations from human populations, these data continued to suggest that CD4 T-cells, and not CD8 T-cells, play a key role in controlling the persistent pulmonary *C. neoformans* infection.

Given individuals with cryptococcal meningitis and advanced HIV have low CD4 T-cell counts, we hypothesized that CD4-depletion of UgCl223-infected mice would result in loss of infection control, increased *C. neoformans* CFUs in the lung, uncontrolled systemic dissemination to the brain, meningitis and ultimately mouse mortality. We first attempted to test this hypothesis using CD4 monoclonal antibody depletion. However, in mice infected with UgCl223 we were unable to achieve the necessary ≥98% CD4 T-cell depletion to sufficiently ablate the lung CD4 T-cell population (**Supplementary Figure 2**). As a result, we were unable to use monoclonal antibody depletion in the context of the persistent UgCl223 infection to examine the role of CD4 T-cells in the lungs.

**Figure 6:**
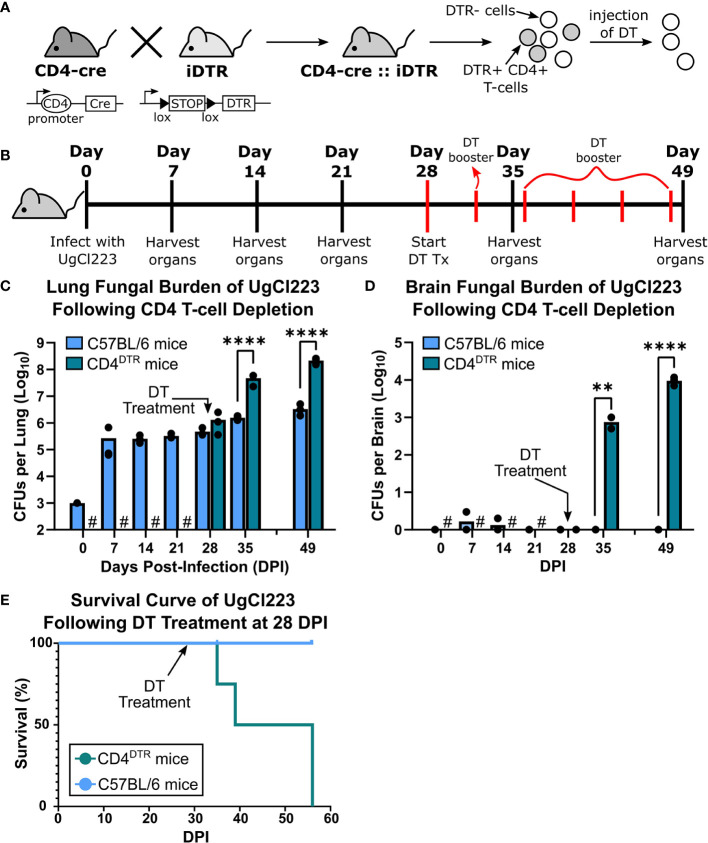
Diphtheria toxin (DT) treatment ablated CD4+ T-cells in CD4^DTR^ mice and caused lethal UgCl223 infections. **(A)** Scheme showing generation of CD4^DTR^ mice and CD4+ T-cell ablation. A cross of CD4-cre mice to iDTR mice generate mice that contain CD4 T-cells sensitive to diphtheria toxin (DT). Filled triangles = loxP sites; arrows = transcriptional activity; open oval = promoter. **(B)** Experimental timeline showing CD4^DTR^ and C57BL/6 mice were intranasally infected with UgCl223 and treated with DT starting at 28 days post-infection. Mice received a booster dose of DT every 4 days to maintain CD4 depletion. **(C)** Lungs and **(D)** brain were analyzed for CFUs at 0-, 7-, 14-, 21-, 28-, 35- and 49-days post-infection. # = no CFUs were determined for timepoints prior to 28-days post-infection for DT-treated CD4^DTR^ mice. Black arrows indicate start of DT treatment. n = 3 mice per timepoint per group. **(E)** Mice were monitored for signs of morbidity and sacrificed at natural endpoint (20% total weight loss, 1g/day weight loss for 2 consecutive days, or neurological symptoms including loss of sternal recumbency, partial paralysis, seizure, convulsion, or coma). n = 4-5 mice per group. For CFU analysis, **p < 0.001, ****p < 0.00001 by two-way ANOVA. For survival kinetics, p=0.0075 by log-rank test.

As an alternative strategy to deplete CD4 T-cells in the lungs during UgCl223 infection, we mimicked the loss of CD4-expressing T-cells observed in HIV by crossing CD4-Cre and Cre-inducible diphtheria toxin receptor (iDTR) mice to generate CD4^DTR^ mice (Buch et al., 2005) (**Figure 6A**). When treated with diphtheria toxin (DT), C57BL/6 mice and iDTR mice had no changes in CD4 T-cell numbers whereas the CD4^DTR^ mice exhibited ≥98% CD4 T-cell ablation both systemically (Buch et al., 2005) and in the lungs (**Supplementary Figure 3**). To determine the role of CD4-expressing T-cells in the UgCl223 infections, CD4^DTR^ mice and control C57BL/6 mice were injected with DT intraperitoneally at 28 days post-infection and received booster DT doses every 4 days (**Figure 6B**). The UgCl223-infected control mice were unaffected by DT treatment up to 55 days post-infection (**Figures 6C–E**). Upon DT treatment, the CD4^DTR^ mice infected with UgCl223 developed significantly higher CFUs in the lungs (**Figure 6C**) and the infection rapidly disseminated systemically to the brain and resulted in up to a 10,000-fold increase in CFUs (**Figure 6D**). All DT-treated CD4^DTR^ mice reached endpoint criteria within 30 days of DT treatment, whereas all infected control mice exhibited no signs of disease or mortality (**Figure 6E**).

**Figure 7:**
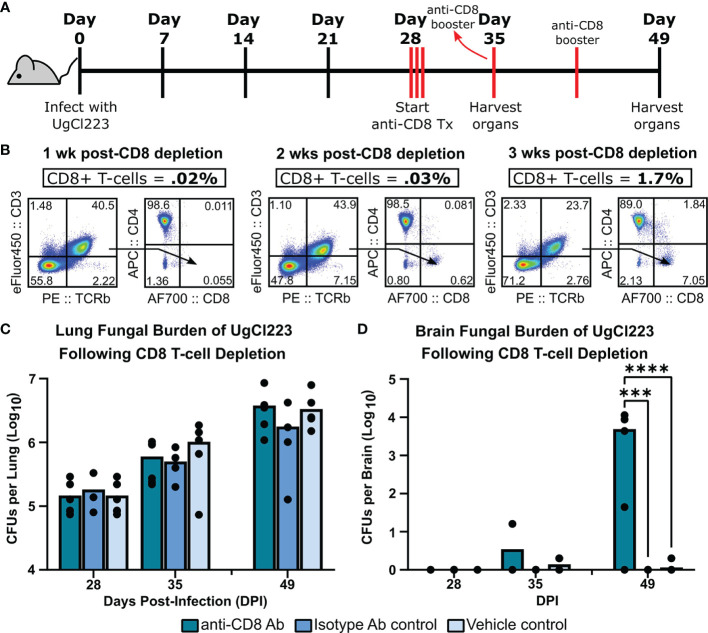
CD8 T-cells were not required to control lung fungal burden during UgCl223 infection. **(A)** Experimental timeline showing C57BL/6 mice were intranasally infected with UgCl223 and treated with 15 mg/kg CD8 monoclonal antibody (2.43) starting at 28 days post-infection for 3 consecutive days and then weekly with booster injections. **(B)** Representative flow cytometric plots showing CD4+CD8- and CD4-CD8+ T-cells isolated from the lungs of infected mice at 1-week post-CD8 depletion (left), 2-weeks post-CD8 depletion (middle), and 3-weeks post-CD8 depletion (right). The gating strategy of the flow cytometric plots was doublet exclusion, gating on live cells, gating on CD3+/TCRβ+ cells, then gating on CD4+CD8- T-cells and CD4-CD8+ T-cells. Frequency of CD3+TCRβ+CD4-CD8+ T-cells was determined by calculating the percentage of CD3+TCRβ+CD4-CD8+ T-cells out of the grandparent gate (i.e., live singlet lymphocytes). **(C)** Lungs and **(D)** brain were analyzed for CFUs at 28-, 35-, and 49-days post-infection. n = 4-5 mice per timepoint per group. ***p < 0.0001, ****p < 0.00001 by two-way ANOVA.

CD8 off-target depletion was previously noted in CD4^DTR^ mice (Buch et al., 2005). We confirmed that CD8 T-cell depletion occurs in the lungs of CD4^DTR^ mice upon DT treatment (**Supplementary Figure 3**), and thus could confound our CD4 T-cell results from the CD4^DTR^ mouse studies. However, we were able to achieve ≥98% CD8 T-cell depletion in the UgCl223 infections using anti-CD8 monoclonal antibody treatment (**Figures 7A, B**) so used this method to analyze the impact of CD8 T cell depletion during UgCl223 infection. We found that CD8 T-cell depletion had no effect on lung fungal burden in the infected mice (**Figure 7C**). Surprisingly, the CD8 depletion did result in increased brain CFUs at 3 weeks post-depletion (**Figure 7D**).

Combined, these data show that CD4 T-cells are important for controlling lung fungal burden, are involved in preventing systemic dissemination and ultimately mortality during UgCl223 infection. In addition, while the CD8 T-cells are dispensable for controlling the pulmonary UgCl223 infection, they likely act in conjunction with the CD4 T-cells either to prevent systemic dissemination or to control the brain infection.

### T_H_1 Cells Are the Predominant Effector CD4 T-Cell Subset Generated During Persistent UgCl223 Infections

**Figure 8:**
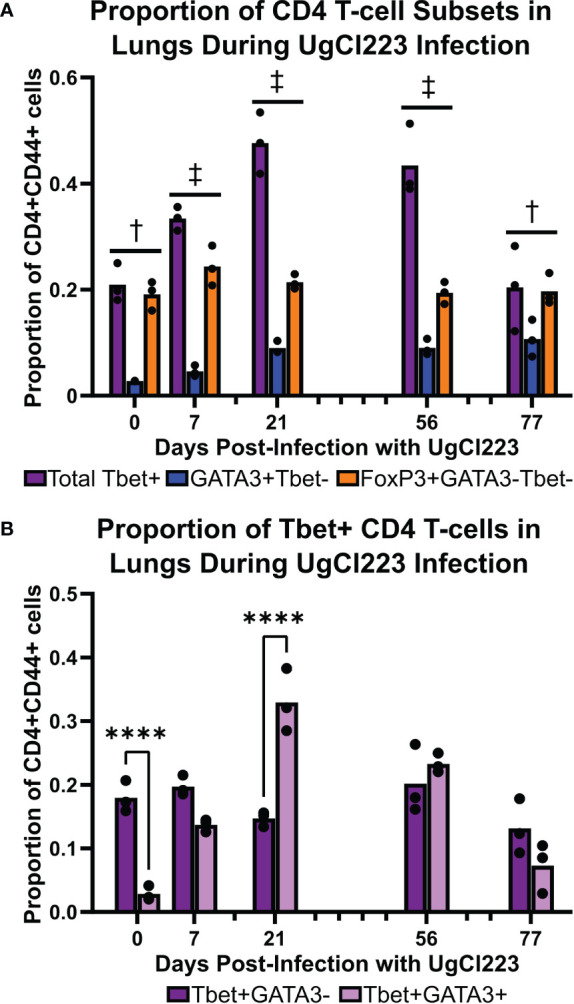
Tbet+ T_H_1 cells were the predominant CD4 T-cell subset in the lungs during the majority of the UgCl223 infection. **(A)** Flow cytometric analysis showing the proportions of CD4+CD44+Tbet+FoxP3- (T_H_1), CD4+CD44+FoxP3+ (T_REG_), and CD4+CD44+GATA3+FoxP3- (T_H_2) cells over time at 0-, 7-, 21-, 56-, and 77-days post-infection. n=3 mice per timepoint. Statistical analysis performed by 2-way ANOVA with Bonferroni correction. † denotes P<0.05 for Tbet+ vs. GATA3+Tbet- and GATA3+Tbet- vs. FoxP3+GATA3-Tbet-. ‡ denotes P< 0.0001 for Tbet+ vs. GATA3+Tbet-; P < 0.05 for Tbet+ vs. FoxP3+GATA3-Tbet-; and P < 0.001 for GATA3+Tbet- vs. FoxP3+GATA3-Tbet-. **(B)** Flow cytometric analysis showing the proportions of CD4+CD44+Tbet+GATA3-FoxP3- and CD4+CD44+Tbet+Gata3+FoxP3- cells over time at 0-, 7-, 21-, 56-, and 77- days post-infection. ****p < 0.0001 by 2-way ANOVA with Bonferroni correction.

We previously showed that the lethal KN99α infection is associated with a non-protective T_H_2 CD4 T-cell response (Wiesner et al., 2015). Unlike during lethal KN99α infection, our data shows that mice infected with the clinical strains UgCl223 and UgCl552 can survive for extended periods of time. Clinical studies suggest that IFNγ, a cytokine associated with immune responses mediated by T_H_1 cells, is beneficial and can be protective against infection (Siddiqui et al., 2005; Jarvis et al., 2012). Therefore, we hypothesized that infection with UgCl223 is associated with a protective T_H_1 CD4 T-cell response. We found that T_H_1 (CD4+ CD44+ FoxP3- Tbet+) cells were the predominant CD4 T-cell subset during UgCl233 infection, ranging from 20% - 48% of activated CD4 T-cells (**Figure 8A**). The proportion of T_H_2 (CD4+ CD44+ FoxP3- Tbet- GATA3+) cells was significantly lower than the T_H_1 cells, and ranged from 4% - 11% of the activated CD4+ T-cells in the UgCl223 infection. Regulatory T-cells (T_REG_, CD4+CD44+FoxP3+) ranged from 20% - 25% of activated CD4+ T-cells during UgCl223 infection (**Figure 8A**) and, unlike in lethal KN99α infections (Wiesner et al., 2016), were present at significantly lower levels compared to the predominant T_H_1 effector cell populations at all but the 0- and 77- day timepoints.

**Figure 9:**
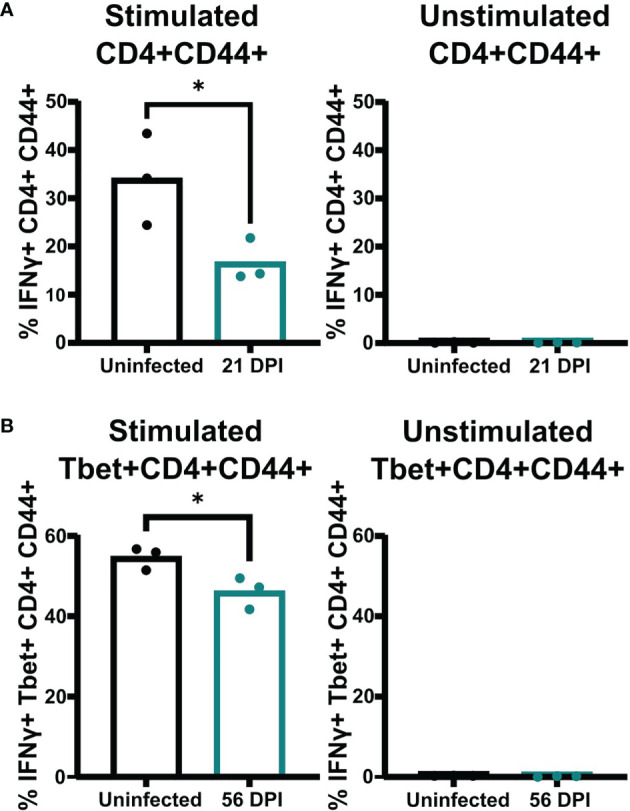
Pulmonary CD4 T-cells isolated from UgCl223 infected mice had diminished IFNγ production upon restimulation. **(A)** The proportion of IFNγ+ CD4+CD44+ cells in the total CD4+CD44+ cell population (%). PMA/ionomycin-stimulated CD4+CD44+ (left) and unstimulated CD4+CD44+ (right) cells were isolated from single cell suspensions of lungs harvested from C57BL/6 mice that were intranasally infected with UgCl223 (n=3) at 21-days post-infection versus mice that were uninfected (n=3) and analyzed by flow cytometry. **(B)** The proportion of IFNg+Tbet+CD4+CD44+ cells in the total Tbet+CD4+CD44+ cell population (%). Following cell sorting, Tbet-zsGreen+ cells from UgCl223-infected Tbet-zsGreen FoxP3-RFP mice (n=3) at 56-days post-infection and from uninfected mice (n=3) were stimulated with PMA/ionomycin (left) or were untreated (right), and then analyzed by flow cytometry. *p < 0.05 by two-tailed t-test.

A previous study showed mice infected with a genetically engineered *C. neoformans* strain expressing IFNγ can clear the infection (Wormley et al., 2007). However, our data showed that mice persistently infected with the *C. neoformans* clinical strains UgCl223 and UgCl552 were unable to clear the infection, and instead maintained stable lung fungal burdens. Upon closer analysis of the Tbet+ T_H_1 cell population, we found a subset of cells that were Tbet+GATA3+ double-positive that ranged from 13% - 33% of the activated CD4+ T-cells, and that represented a significantly higher proportion of the population at 21 days post-infection compared to the Tbet+GATA3- cells (**Figure 8B**). Stable Tbet+GATA3+ double positive CD4 T-cell populations were observed during parasitic infections and had decreased IFNγ production upon restimulation (Peine et al., 2013; Bouchery et al., 2014; Bock et al., 2017). Therefore, we questioned whether the IFNγ-mediated effector function of the T_H_1 cells remained intact throughout the UgCl223 infection. When we isolated activated CD4+CD44+ T-cells from the lungs of mice infected with UgCl223, restimulated the cells with PMA/ionomycin, and then measured their IFNγ production, we found that the activated CD4+CD44+ T-cells had significantly diminished IFNγ production compared to cells from uninfected control mice (**Figure 9A**). As expected, the CD4+CD44+ T-cells from both the uninfected controls and the UgCl223 infected mice did not produce IFNγ without PMA/ionomycin restimulation (**Figure 9A**). To specifically test our hypothesis that an impaired T_H_1 response is the cause of this diminished IFNγ production by the CD4 T-cell population, we isolated CD4 T-cells from lungs of Tbet-zsGreen FoxP3-RFP mice infected with UgCl223 and sorted for Tbet-zsGreen+ FoxP3-RFP- CD4+CD44+ T-cells (**Supplementary Figure 5**). Following PMA/ionomycin restimulation, the Tbet-zsGreen+FoxP3-RFP- CD4+CD44+ T-cells had significantly diminished IFNγ production compared to cells from uninfected control mice (**Figure 9B**). Altogether, these data show that the T_H_1 cells are the predominant CD4 T-cell subset produced during persistent UgCl223 infection, but IFNγ production by these T_H_1 cells is impaired.

## Discussion

In the current study, we address two issues that have hindered progress in understanding the host immune response to latent cryptococcosis: 1) the lack of defined criteria to assess latent cryptococcosis in animal models and 2) the limited genetic and immunological tools available to study the host immune response in non-murine models of latent cryptococcosis. We used clinical (Baker, 1976; Dromer et al., 2010; Alanio, 2020; Pirofski and Casadevall, 2020) and experimental (Goldman et al., 1994; Goldman et al., 2000) properties of latent cryptococcosis to propose a set of standardized criteria to define latency in animal models of persistent *C. neoformans* infection, as follows: no clinical signs of disease throughout the entirety of infection, continuous increases in the animal’s weight, stable fungal counts in the lungs throughout the entirety of the infection, generation of pulmonary granulomas with no alveolar inflammation in the surrounding lung parenchyma, serum CrAg LFA negative, and no animal mortality. We then assessed whether a novel mouse model of persistent *C. neoformans* infection using the clinical isolates UgCl223 and UgCl552 met these criteria for latency. We found that a persistent infection could be established in C57BL/6 mice *via* low-dose inhalation of the *C. neoformans* clinical isolates, which were from individuals with advanced HIV and cryptococcal meningitis. The infected mice gained weight, had no clinical signs of disease, had stable fungal burdens, had yeast cells contained within pulmonary granulomas, and had predominantly healthy lung parenchyma tissue with minimal inflammation. While a majority of mice infected with UgCl223 survive, some mortality was observed – typically between 70-80 days post-infection. This mortality appears to be due to an inability to fully establish the “latent” stage of the infection, because the surviving mice can live longer than 270 days with no evidence of clinical disease (Ding and Nielsen, unpublished data).

In the persistent rat infection model, serum GXM was detected *via* latex agglutination (LA) and enzyme-linked immunosorbent assay (ELISA) by 10 days post-infection (Goldman et al., 1994). However, in a later study, the same researchers did not detect serum GXM *via* ELISA throughout the entirety of the infection (Goldman et al., 2000). It is not clear why there is such a large discrepancy between the two separate studies. Interestingly, LA tests have been found to be more sensitive compared to ELISAs for detecting GXM in non-HIV cryptococcosis patients with low antigenemia (Panackal et al., 2014). Thus, it is feasible that in the second study, 52D-infected rats had a serum GXM load that was below the ELISA level of detection. LFAs have much higher sensitivity and specificity in detecting serum GXM, or cryptococcal antigen (CrAg), compared to commercially available ELISA (Tanner et al., 1994) and LA tests (Boulware et al., 2014). Therefore, while CrAg titers were detected in UgCl223 infected mice at 70 days post-infection, it is important to note that the majority of serum CrAg titers were very low - ranging from 1:2 to 1:5 – and below levels that would have been detected in the rat model. There were two UgCl223 infected mice that had CrAg titers of 1:20, but both mice did not have any visible signs of disease and one mouse survived an additional week before it was euthanized due to a non-healing lesion that was not associated with the latent *C. neoformans* infection. We also identified one mouse that was CrAg positive at 1:2 at 200 days post-infection, but was later found to be CrAg negative at 230 days post-infection (Ding and Nielsen, unpublished data). In contrast, we detected a CrAg titer of 1:2560 in a KN99α-infected mouse that had developed neurological symptoms of cryptococcal meningitis and was subsequently sacrificed due to reaching endpoint criteria. Interestingly, our findings mirror clinical studies which show that very low CrAg titers are poor indicators of the potential to develop cryptococcal meningitis, whereas high CrAg titers are more accurate in predicting active disease (Dubbels et al., 2017; Harrington et al., 2021). Given that positive serum CrAg titers have occasionally been detected in immunocompetent individuals (Casadevall and Perfect, 1998), it is possible that LFAs have the sensitivity required to detect latent *C. neoformans* infections in individuals with no prior history of cryptococcal disease *via* low CrAg titers. Overall, CrAg LFAs remain a useful diagnostic tool and have the potential to unravel nuances of latent *C. neoformans* infections that we have yet to appreciate.

We did observe extrapulmonary dissemination in the brain (at 70 days post-infection) and spleen (at 35 days post-infection) during infection with both UgCl223 and UgCl552. However, this dissemination was only observed in a subset of mice and the mice exhibited no neurological symptoms or poor health when they had detectable brain CFUs. Similarly, CFUs in the kidneys, spleen, and brain are observed as early as 7 days post-infection in rats infected with *C. deneoformans* 52D (Goldman et al., 1994). Importantly, in humans the cervical lymph nodes and the prostate have also been identified as extrapulmonary reservoirs for latent *C. neoformans* infection (Baker, 1976; Larsen, 1989; Bao et al., 2013; Gurung et al., 2014). This evidence led us to question the dogma, initially derived from other systems, that latent pulmonary infections do not disseminate (Brunet et al., 2018). We concluded, based on the evidence for extrapulmonary dissemination in latent infections of healthy humans, that absence of extrapulmonary dissemination should not be a criterion for animal models of latent cryptococcosis.

While the pathogenesis of *C. neoformans* is distinct from that of *M. tuberculosis*, similarities between the two pathogens such as the initial pulmonary infection and granulomatous inflammation could suggest that criteria for latency in *M. tuberculosis* animal models can be used for *C. neoformans* latency (Pirofski and Casadevall, 2020). In murine models of latent tuberculosis, the criteria for latency involve low-dose, persistent infections with few granulomas, low intensity alveolar inflammation, continuous increases in animal weights, stable bacterial counts in the lungs, and no clinical signs of disease or spontaneous relapse (Shi et al., 2011). Clearly, our criteria for latent cryptococcosis in animal models closely follows that of latent tuberculosis mouse models. The concept of subclinical infections has been introduced in latent tuberculosis studies using cynomolgus macaques – asymptomatic monkeys had positive *M. tuberculosis* cultures *via* bronchoalveolar lavage or gastric aspirate several months to years after infection (Lin et al., 2009). Under this strict definition of latency (i.e., the absence of positive cultures), our novel murine model of persistent *C. neoformans* infection could be considered subclinical. However, we do not think the subclinical categorization of our mouse model detracts from its value in providing insight into host mechanisms of control against pulmonary cryptococcosis. Altogether, our data provides strong support that mice infected with the clinical *C. neoformans* strains, UgCl223 and UgCl552, establish a persistent and stabilized infection that can be considered at most latent, or at least subclinical.

While infection with UgCl223 and UgCl552 in mice may not replicate all aspects of human latent *C. neoformans* infection, the potential benefits of this model are many. The use of *C. neoformans* (serotype A, var. *grubii*) clinical isolates allows more accurate comparisons between the mouse latent infection model and the reactivation of latent infections that occurs in the vast majority of human patients (De Sousa et al., 2021). Although C57BL/6 mice have a truncated CXCL11 gene (Groom and Luster, 2011), we observed very similar infection trajectories between C56BL/6 mice and A/JCr mice with only subtle differences between the two inbred backgrounds (Mukaremera et al., 2019). The UgCl552 strain was more frequently cleared in A/JCr mice, compared to C57BL/6 mice. In contrast, infection with UgCl223 did not result in significant differences between the two mouse backgrounds. Thus, we chose the UgCl223 strain to carry forward for the bulk of our analysis. These similarities and differences between the mouse backgrounds and clinical isolates highlight a critical issue with extrapolating our results more broadly. We anticipate the model that we developed will allow us to probe critical interactions between the host immune response and the *C. neoformans* pathogen in the context of the latent infection. Yet, how specific these interactions will be to the C57BL/6 inbred mouse immune response and to the antigenic profile of UgCl223 is an important question that will need to be addressed in future experiments. Even if specificity is uncovered, it is highly likely that there will be a generalized set of factors, which will be independent of the host background, and necessary to control the latent infection; this can be uncovered using the widely used C57BL/6 mouse background in which many mouse genetic knock-out strains are already available.

Interestingly, we found that while total lung immune cell numbers increased significantly over time during the mouse *C. neoformans* latent infection, the relative frequencies of each immune cell type remained stable. In addition, the gradual increase in pulmonary leukocytes we observed suggests that stabilization of the latent infection may occur over an extended period of time; this hypothesis is supported by the observation that pulmonary granulomas are not visible until 14 days post-infection and changes in the granuloma morphology can be observed up to 70 days post-infection. Unlike in the lethal infection, the latent infection was not characterized by significantly increased recruitment of eosinophils to the lung parenchyma. Eosinophils are known to recruit and promote the differentiation of effector CD4 T-cells to the T_H_2 cell phenotype (Spencer and Weller, 2010). Our previous studies showed that T_H_2 cells are the predominant CD4 T-cell subset during lethal infection and enhance disease progression (Wiesner et al., 2015). However, the absence of pulmonary eosinophils in the latent infection does not result in a T_H_2-dominant response, thereby prolonging the survival of the host. Indeed, our data show that the dominant CD4 T-cell subset generated during latent infection is T_H_1 cells.

Upon PMA/ionomycin restimulation, the activated pulmonary effector CD44+ CD4 T-cells isolated from latently infected mice had diminished IFNγ production compared to the same cells isolated from uninfected control mice. It is not clear what is causing this diminished capacity to produce IFNγ. In the lethal *C. neoformans* infection, T_REG_ cell numbers increase dramatically and these T_REG_ cells specifically suppress the dominant T_H_2 cell proliferation (Wiesner et al., 2016). We did not see an increase in the percentage of T_REG_ cells throughout the course of the latent infection that was concomitant with the increase in the T_H_1 cell population, thus it seems unlikely that the T_REG_ cells are dampening the T_H_1 cell IFNγ production. Another possibility is immune exhaustion that may arise from chronic exposure to *C. neoformans*. However, previous studies using the 52D persistent mouse model showed treatment with anti-PD1 antibody did not enhance IFNγ expression (Roussey et al., 2017). Another possible cause for the decrease in IFNγ production could be the production of the Tbet+GATA3+ double positive T_H_1 cells we observed during the latent infection. Stable Tbet+GATA3+ double positive CD4 T-cell populations are produced during parasitic infections and have low IFNγ production upon restimulation (Peine et al., 2013; Bouchery et al., 2014; Bock et al., 2017). Since IFNγ was shown to promote clearance of *C. neoformans* during clinical trials (Siddiqui et al., 2005; Jarvis et al., 2012) and mice infected with genetically engineered *C. neoformans* strains expressing IFNγ can clear the infection (Wormley et al., 2007), the diminished IFNγ production could indicate that the activated CD4 T-cells have impaired capacity to completely clear the pulmonary infection, ultimately promoting the persistence of the latent *C. neoformans* infection in the lungs.

Development of cryptococcal meningitis is largely seen in the context of CD4 T-cell deficiency, such as advanced HIV infection. In this mouse latent infection model, we found that CD4 T-cells were important for control of the pulmonary infection and mirrored clinical findings in advanced HIV patients with low CD4 T-cell counts that develop cryptococcal meningitis. Depletion of T-cells that express CD4 in infected mice resulted in a rapid increase in lung and brain fungal burden that ultimately led to lethal meningitis. Importantly, we had to use CD4^DTR^ mice to deplete the CD4 T-cells in the lungs of latently infected mice and the DT treatment caused off-target depletion of CD8 T-cells. While targeted depletion of the CD8 T-cells using monoclonal antibodies did not increase lung fungal burdens, indicating that the CD8 T-cells are dispensable during control of the latent pulmonary infection, we did see enhanced dissemination to the brain. Given that we have yet to identify a scenario in which we can sufficiently deplete only the lung CD4 T-cells in the context of the latent infection, we cannot conclude that the CD4 T-cells are responsible for control of the latent infection, rather our current data support a scenario in which the CD4 and CD8 T-cells work in concert. CD4 T-cells appear to have a larger role in controlling the latent pulmonary infection while both CD4 and CD8 cells may work together to prevent dissemination.

It is clear that the adaptive immune response, of which CD4 T-cells are an important component, is required for controlling latent pulmonary *C. neoformans* infections in healthy mice. However, differences in lung histopathology, lack of eosinophilia, and the predominant type 1 immune response compared to the lethal infection model indicates that our understanding of how the host immune response controls pulmonary *C. neoformans* infection is still lacking. Future experiments will need to focus on determining both the mechanism by which the T_H_1 cells control pulmonary cryptococcosis and why the host immune system cannot fully eradicate the latent *C. neoformans* infection. In addition, we hope that our proposed criteria for *C. neoformans* latency will serve as a useful tool to refine this or other animal models of latent infection; and that these models are then the foundation for study of this ubiquitous part of the host-pathogen interaction in *C. neoformans* disease.

## Data Availability Statement

The original contributions presented in the study are included in the article/**Supplementary Material**. Further inquiries can be directed to the corresponding author.

## Ethics Statement

The animal study was reviewed and approved by University of Minnesota Institutional Animal Care and Use Committee.

## Author Contributions

MD, DW, and KN conceived and designed the experiments. MD, KS, JN, and KJ performed the experiments. MD, JN, and KN analyzed the data. KN contributed reagents, materials, and analysis tools. MD and KN wrote the paper. All authors contributed to the article and approved the submitted version.

## Funding

This work was supported by the National Institutes of Health [R01AI134636, R01NS118538, and R21AI150303 to KN]. MD was supported by the National Institutes of Health [F30AI155292], a University of Minnesota Medical Student Training Program [T32GM008244], and a University of Minnesota Lung Biology Dinnaken Fellowship. DW was supported by a Center for Immunology Training Program [T32AI007313], a University of Minnesota Department of Microbiology Watson Fellowship, and a University of Minnesota Doctoral Dissertation Fellowship. KJ was supported by the National Institutes of Health [F31AI148047].

## Conflict of Interest

The authors declare that the research was conducted in the absence of any commercial or financial relationships that could be construed as a potential conflict of interest.

## Publisher’s Note

All claims expressed in this article are solely those of the authors and do not necessarily represent those of their affiliated organizations, or those of the publisher, the editors and the reviewers. Any product that may be evaluated in this article, or claim that may be made by its manufacturer, is not guaranteed or endorsed by the publisher.

## Supplementary Material

Supplementary Figure 1 Bulk leukocyte flow cytometry gating strategy. Representative flow scatter plots of single cell suspension isolated from the lungs of a C57BL/6 mouse at 100 days post-infection with strain UgCl223.

Supplementary Figure 2 CD4 monoclonal antibody depletion resulted in partial depletion of pulmonary CD4 T-cells during latent infection. C57BL/6 mice were intranasally infected with UgCl223 and treated with 5 mg/kg, 10 mg/kg, 15 mg/kg, 25 mg/kg, and 50 mg/kg CD4 monoclonal antibody (GK1.5) starting at 28 days post-infection. Representative flow cytometric plots showing CD4+CD8- and CD4-CD8+ T-cells isolated from lungs of infected mice at 1-week post-CD4 depletion (top) and 2 weeks post-CD4 depletion (bottom). The gating strategy of the flow cytometric plots was doublet exclusion, gating on live cells, gating on CD3+/TCRβ+ cells, then gating on CD4+CD8 T-cells and CD4-CD8+ T-cells. Frequency of CD3+TCRβ+CD4+CD8- T-cells was determined by calculating the percentage of CD3+TCRβ+CD4+CD8- T-cells out of the grandparent gate (i.e., live singlet lymphocytes).

Supplementary Figure 3 Diphtheria toxin (DT) treatment ablated CD4+ T-cells in CD4^DTR^ mice. Representative flow scatterplot of lung CD3+TCRβ+CD4+CD8- and CD3+TCRβ+CD4-CD8+ T-cells at 1-, 2-, and 3-weeks post-DT treatment. The gating strategy of the flow cytometric plots was doublet exclusion, exclusion of dead cells, gating on lymphocytes based on size and granularity, gating on CD3+TCRβ cells, then gating on CD4+CD8- and CD4-CD8 T-cells. Frequency of CD3+TCRβ+CD4+CD8- T-cells was determined by calculating the percentage of CD3+TCRβ+CD4+CD8- T-cells out of the grandparent gate (i.e., live singlet lymphocytes).

Supplementary Figure 4 Th subsets flow cytometry gating strategy. Representative flow scatter plots of a single cell suspension isolated from the lungs of a C57BL/6 mouse at 56 days post-infection with strain UgCl223. Fluorescence minus one (FMO) controls were used to determine gating for BV421-Tbet and AF647-GATA3 populations.

Supplementary Figure 5 Experimental workflow and gating strategy for IFNγ restimulation assays. **(A)** Tbet-zsGreen cells were sorted from negatively-selected CD4 T-cells isolated from the lungs of UgCl223-infected Tbet-zsGreen FoxP3-RFP mice, or **(B)** CD4 T-cells were isolated from UgCl223-infected C57BL/6 mice. **(C)** Representative flow scatter plots of a flow sort for Tbet-zsGreen+ cells isolated from UgCl223-infected Tbet-zsGreen FoxP3-RFP mice. **(D)** Representative flow scatter plots for IFNg+CD4+CD44+ cells following PMA/ionomycin restimulation.
