# Supplementary figures and images for: Use of Clinical Isolates to Establish Criteria for a Mouse Model of Latent Cryptococcus neoformans Infection
Source: Front Cell Infect Microbiol. 2022 Feb 2;11:804059. doi: 10.3389/fcimb.2021.804059 (PMC8847453; doi:10.3389/fcimb.2021.804059)

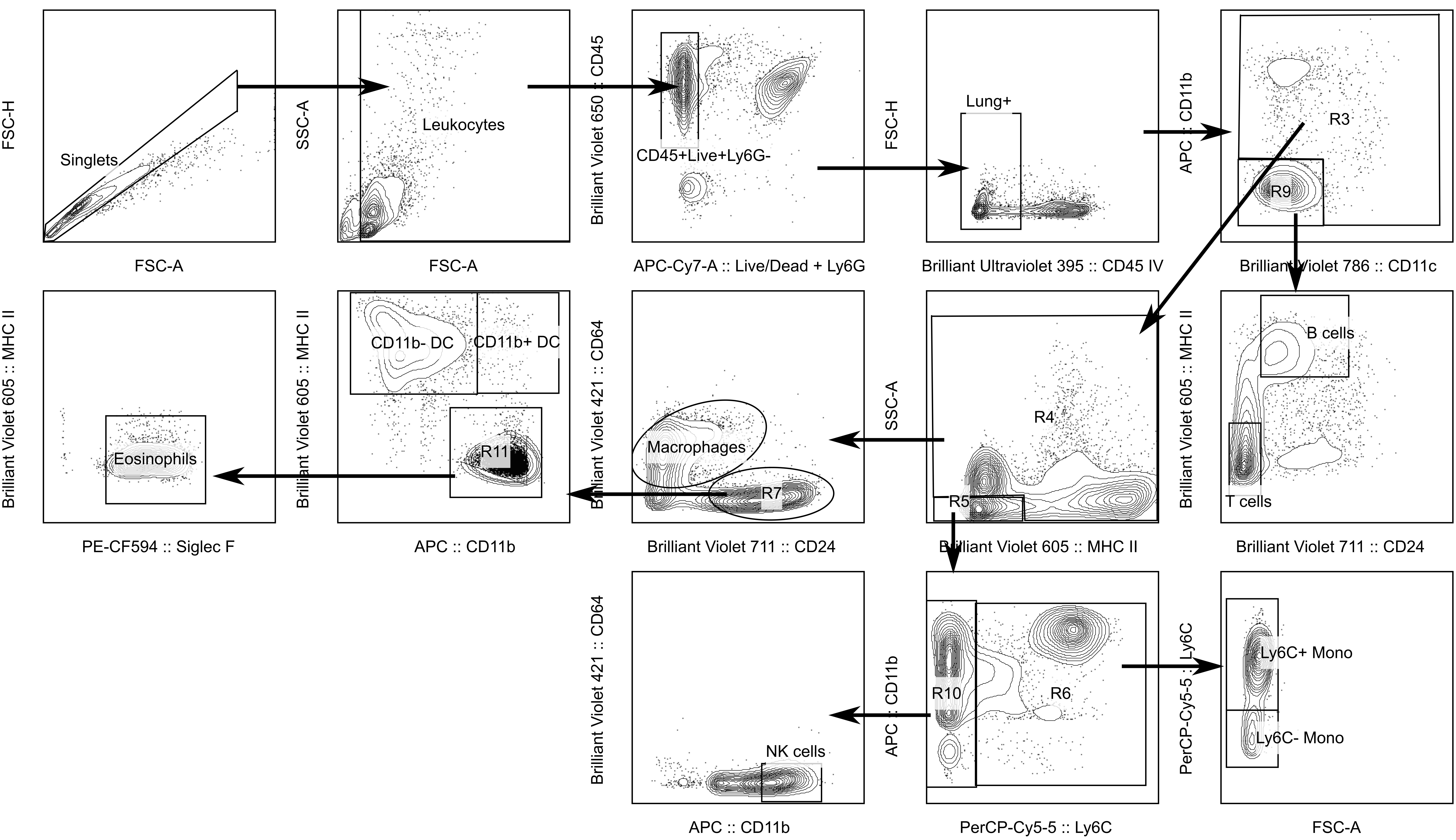

Supplement: Supplementary Figure 1 — Bulk leukocyte flow cytometry gating strategy. Representative flow scatter plots of single cell suspension isolated from the lungs of a C57BL/6 mouse at 100 days post-infection with strain UgCl223. [file Image_1.jpeg]

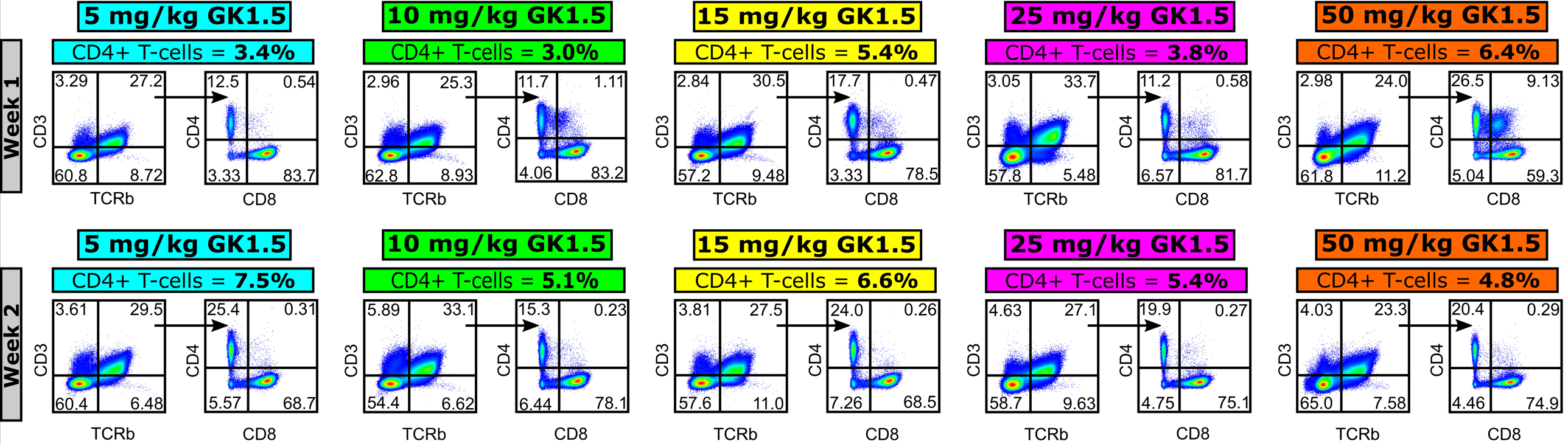

Supplement: Supplementary Figure 2 — CD4 monoclonal antibody depletion resulted in partial depletion of pulmonary CD4 T-cells during latent infection. C57BL/6 mice were intranasally infected with UgCl223 and treated with 5 mg/kg, 10 mg/kg, 15 mg/kg, 25 mg/kg, and 50 mg/kg CD4 monoclonal antibody (GK1.5) starting at 28 days post-infection. Representative flow cytometric plots showing CD4+CD8- and CD4-CD8+ T-cells isolated from lungs of infected mice at 1-week post-CD4 depletion (top) and 2 weeks post-CD4 depletion (bottom). The gating strategy of the flow cytometric plots was doublet exclusion, gating on live cells, gating on CD3+/TCRβ+ cells, then gating on CD4+CD8 T-cells and CD4-CD8+ T-cells. Frequency of CD3+TCRβ+CD4+CD8- T-cells was determined by calculating the percentage of CD3+TCRβ+CD4+CD8- T-cells out of the grandparent gate (i.e., live singlet lymphocytes). [file Image_2.jpeg]

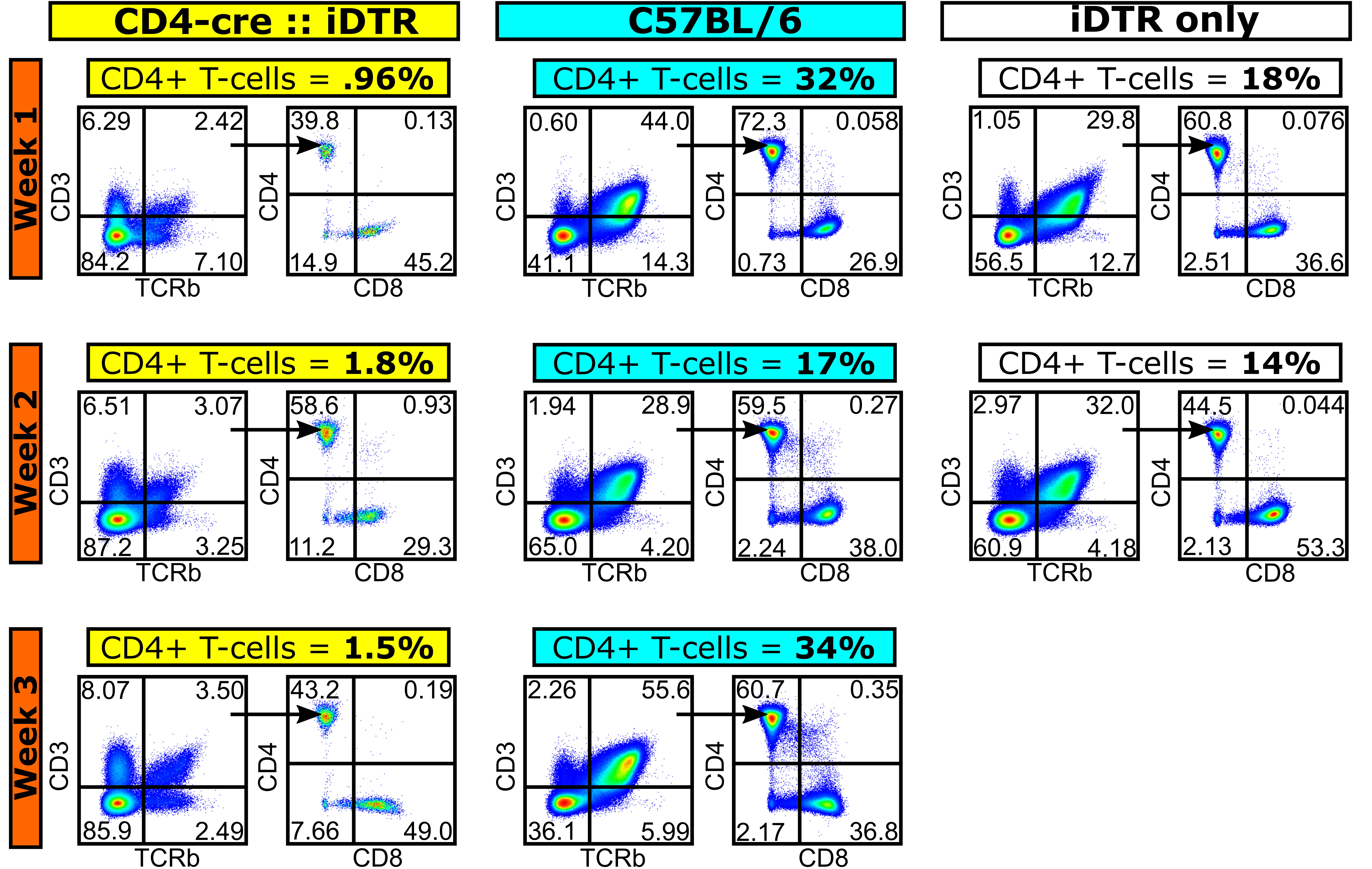

Supplement: Supplementary Figure 3 — Diphtheria toxin (DT) treatment ablated CD4+ T-cells in CD4DTR mice. Representative flow scatterplot of lung CD3+TCRβ+CD4+CD8- and CD3+TCRβ+CD4-CD8+ T-cells at 1-, 2-, and 3-weeks post-DT treatment. The gating strategy of the flow cytometric plots was doublet exclusion, exclusion of dead cells, gating on lymphocytes based on size and granularity, gating on CD3+TCRβ cells, then gating on CD4+CD8- and CD4-CD8 T-cells. Frequency of CD3+TCRβ+CD4+CD8- T-cells was determined by calculating the percentage of CD3+TCRβ+CD4+CD8- T-cells out of the grandparent gate (i.e., live singlet lymphocytes). [file Image_3.jpeg]
